# Organometallic nucleosides induce non-classical leukemic cell death that is mitochondrial-ROS dependent and facilitated by TCL1-oncogene burden

**DOI:** 10.1186/s12943-015-0378-1

**Published:** 2015-06-04

**Authors:** Christian Prinz, Elena Vasyutina, Gregor Lohmann, Alexandra Schrader, Steffen Romanski, Christoph Hirschhäuser, Petra Mayer, Corazon Frias, Carmen D. Herling, Michael Hallek, Hans-Günther Schmalz, Aram Prokop, Dimitrios Mougiakakos, Marco Herling

**Affiliations:** Laboratory of Lymphocyte Signaling and Oncoproteome, Department I of Internal Medicine, Center for Integrated Oncology (CIO) Köln-Bonn, and Excellence Cluster for Cellular Stress Response and Aging-Associated Diseases (CECAD), University of Cologne, Cologne, Germany; Division of Organic Chemistry, University of Cologne, Cologne, Germany; Department of Pediatric Hematology/Oncology, Children’s Hospital Cologne, Cologne, Germany; Department I of Internal Medicine, CIO Köln-Bonn, and CECAD, University of Cologne, Cologne, Germany; Department of Internal Medicine, University of Erlangen, Erlangen, Germany

**Keywords:** CLL, ROS, Organometallic nucleosides, TCL1, Mitochondrial respiration

## Abstract

**Background:**

Redox stress is a hallmark of the rewired metabolic phenotype of cancer. The underlying dysregulation of reactive oxygen species (ROS) is interconnected with abnormal mitochondrial biogenesis and function. In chronic lymphocytic leukemia (CLL), elevated ROS are implicated in clonal outgrowth and drug resistance. The pro-survival oncogene T-cell leukemia 1 (TCL1) is causally linked to the high threshold towards classical apoptosis in CLL. We investigated how aberrant redox characteristics and bioenergetics of CLL are impacted by TCL1 and if this is therapeutically exploitable.

**Methods:**

Bio-organometallic chemistry provided compounds containing a cytosine nucleobase, a metal core (ferrocene, ruthenocene, Fe(CO)_3_), and a 5**’**-CH2O-TDS substituent. Four of these metal-containing nucleoside analogues (MCNA) were tested for their efficacy and mode of action in CLL patient samples, gene-targeted cell lines, and murine TCL1-transgenic splenocytes.

**Results:**

The MCNA showed a marked and selective cytotoxicity towards CLL cells. MCNA activity was equally observed in high-risk disease groups, including those of del11q/del17p cytogenetics and of clinical fludarabine resistance. They overcame protective stromal cell interactions. MCNA-evoked PARP-mediated cell death was non-autophagic and non-necrotic as well as caspase- and P53-independent. This unconventional apoptosis involved early increases of ROS, which proved indispensible based on mitigation of MCNA-triggered death by various scavengers. MCNA exposure reduced mitochondrial respiration (oxygen consumption rate; OCR) and induced a rapid membrane depolarization (∆ΨM). These characteristics distinguished the MCNA from the alkylator bendamustine and from fludarabine. Higher cellular ROS and increased MCNA sensitivity were linked to TCL1 expression. The presence of TCL1 promoted a mitochondrial release of in part caspase-independent apoptotic factors (AIF, Smac, Cytochrome-c) in response to MCNA. Although basal mitochondrial respiration (OCR) and maximal respiratory capacity were not affected by TCL1 overexpression, it mediated a reduced aerobic glycolysis (lactate production) and a higher fraction of oxygen consumption coupled to ATP-synthesis.

**Conclusions:**

Redox-active substances such as organometallic nucleosides can confer specific cytotoxicity to ROS-stressed cancer cells. Their P53- and caspase-independent induction of non-classical apoptosis implicates that redox-based strategies can overcome resistance to conventional apoptotic triggers. The high TCL1-oncogenic burden of aggressive CLL cells instructs their particular dependence on mitochondrial energetic flux and renders them more susceptible towards agents interfering in mitochondrial homeostasis.

**Electronic supplementary material:**

The online version of this article (doi:10.1186/s12943-015-0378-1) contains supplementary material, which is available to authorized users.

## Introduction

The current therapeutic challenges in cancer, including chronic lymphocytic leukemia (CLL) the most prevalent leukemia of adults in the western world, involve the targeting of tumor-specific pathways in a more profound fashion than accomplished by conventional cytostatics [1]. In CLL, chemo-immunotherapies with nucleosides like fludarabine in combination with antibodies, have significantly improved response rates [2], but the majority of patients eventually relapse due to incomplete clonal eradication and finally develop refractory disease. A major underlying reason for such treatment failures are resistances of the leukemic (sub)clones towards drug-induced triggering of classical apoptosis [3]. Mediators of such protection in CLL are a marked pro-survival impact by micro-environmental niches [4] and genetic deficiencies to evoke an adequate p53 mediated apoptotic response. The latter is particularly found in the clinically high-risk subsets of 11q23/ATM or 17p/TP53 deleted/mutated CLL [5, 6].

A key to overcome such high thresholds for classical apoptosis would be to exploit independent forms of (programmed) cell death. Such therapeutic strategies would bypass major modes of resistance to most currently used substances. We previously identified organochalcogens (organoselenium, -tellurium compounds) to act as ‘sensor/effector’ catalysts of reactive oxygen species (ROS), particularly in a specific tumor-to-normal cell fashion across various cancer cell types, including CLL [7, 8]. These substances exploited the aberrant redox equilibrium of enhanced radical production and reduced glutathione (GSH) buffer levels in CLL cells as their selective vulnerability by increasing the elevated ROS levels towards a cytotoxic threshold. The therapeutic potential of modulating ROS in CLL had been demonstrated by others as well [9, 10] and this can be particularly efficient when mitochondrial respiration is simultaneously inhibited [11]. Encouragingly, ROS-mediated induction of CLL cell apoptosis was shown to be independent of p53-functional status [12].

Elevated levels of ROS, the byproduct of normal cell respiration, are a hallmark of the rewired metabolic cancer phenotype [13]. Due to their genotoxic effects and messenger function in milieu-derived growth signaling, especially via the B-cell receptor (BCR) [14, 15], ROS are implicated in transformation, clonal sustenance, and drug resistance in CLL particularly in advanced disease and after previous therapy [16]. Protective stromal cells provide cystine for anti-oxidant GSH synthesis to CLL cells and thereby relieve their ROS stress [17].

A central oncogenic mechanism in CLL is overexpression of the adapter molecule T-cell leukemia 1 (TCL1). Mice transgenic (tg) for human TCL1 driven by the Eμ immunoglobulin (IG) gene enhancer (Eμ-TCL1) model human CLL with most fidelity to its aggressive IGHV gene unmutated subset [18]. Through a physical interaction with the AKT growth kinase, TCL1 enhances proximal milieu-derived signaling, particularly acting as a sensitizer for BCR-triggered cellular fates [19]. High-level TCL1 is associated with high-risk disease features and poorer therapeutic outcome [19, 20].

These data provide strong rationales to therapeutically exploit ROS as mediators of non-classical cell death pathways in CLL in the context of their notorious resistance to apoptosis, especially linked to high TCL1 expression. We therefore designed novel metal-containing nucleoside analogues (MCNA) and present here their efficient and selective cell death induction in CLL. This action was indiscriminate of cytogenetic risk subsets and irrespective of protective stromal cell contact. Their non-autophagic and non-necrotic cytotoxic activity involved an early ROS induction and was independent of p53 or caspase activation. We link the oncogenic impact of TCL1 to elevated ROS and altered mitochondrial energetic flux, which results in an enhanced sensitivity to redox active agents, e.g. MCNA, representing a potent vulnerability.

## Results

### Chemistry and selection of the novel organometallic nucleoside analogues

**Fig. 1:**
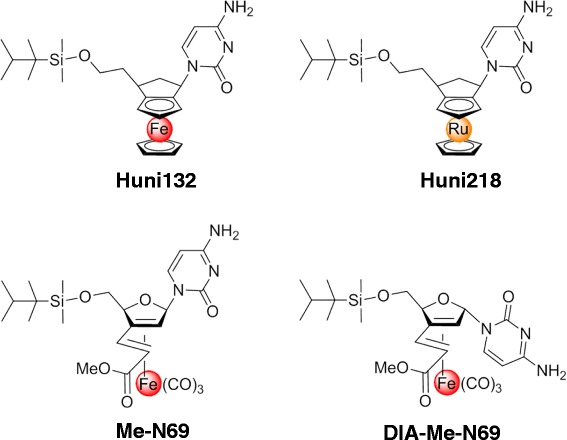
Chemical structure of the selected novel metal-containing nucleoside analogues. A panel of metal-containing nucleosides was designed and synthesized, with the 4 illustrated compounds selected for this study. Huni132 is a ferrocene based nucleoside analogue, Huni218 is the corresponding ruthenium congener. Me-N69 is a butadiene Fe(CO)_3_-complex and Dia-Me-N69 is formed as a diastereomeric byproduct during the synthesis of Me-N69

We have been extensively studying butadiene Fe(CO)_3_-complexes like Me-N69 and could show that their iron-fragment is crucial for the cell death inducing activity in BJAB lymphoma cells [21]. We found cytosine to be the nucleobase most effective for cell death induction as well as a non-polar protecting group at O5’ to be essential for this substance efficacy [21]. There was no significant difference in activity between furanoid compounds (partially hydrated furanes) like Me-N69 and its carbocyclic congeners [21]. Given these observations, we designed optimized MCNA. They were synthesized according to our established protocols [21–23]. Based on chemical properties and extrapolated activity indices, 4 compounds were further selected for this study (Fig. 1). Huni132 is a ferrocene-based nucleoside analogue, which contains Fe(II). Huni218 is the corresponding ruthenium congener. Me-N69 is a butadiene Fe(CO)_3_-complex, which contains Fe(O) and can donate up to three electrons upon oxidation. Huni132, Me-N69, and Huni218 resemble natural nucleosides with respect to the relative position of the cytosine unit and the 5’-side chain. Dia-Me-N69 is formed as a diastereomeric byproduct during the synthesis of Me-N69. It features a stereochemically inverse, hence ‘atypical’, configuration at C1 and was added as a control.

### The novel organometallic nucleoside analogues induce marked in-vitro CLL cell death irrespective of the presence of high-risk disease determinants or stromal cell protection

**Fig. 2:**
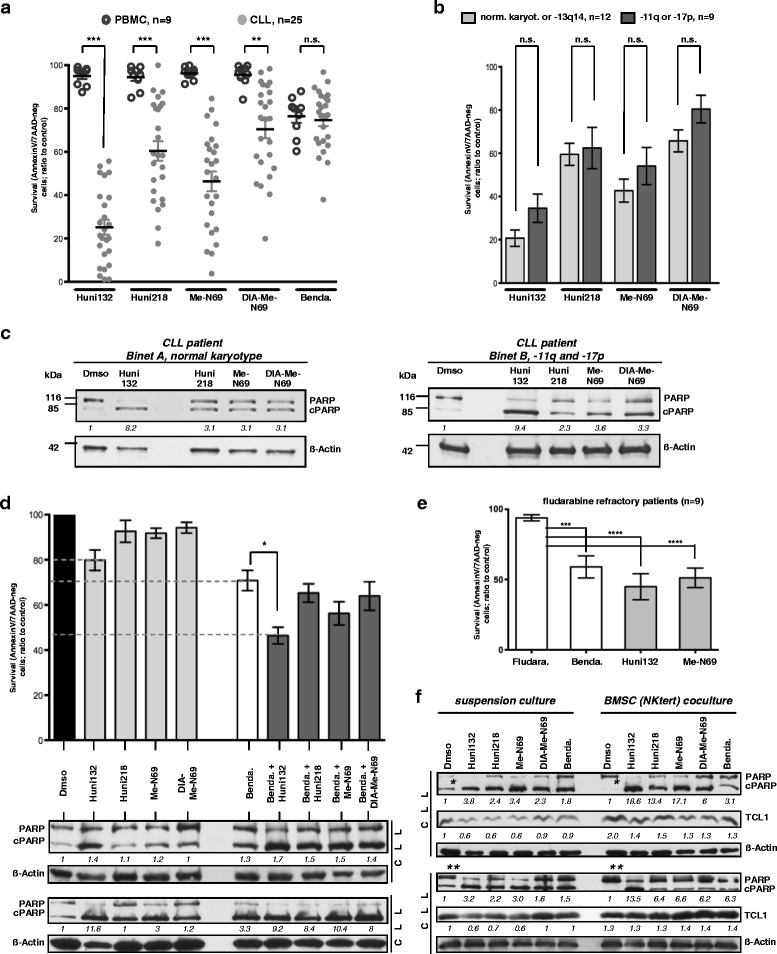
The novel organometallic nucleoside analogues (MCNA) induce marked in-vitro cell death across all cytogenetic and clinical risk subsets of CLL and can overcome stromal cell protection. Incubation (24 h) of primary CLL suspension cultures (n = 25) or healthy-donor derived PBMC (n = 9) with MCNA (10 μM) or bendamustine. Cytotoxicity as per AnnexinV/7AAD flow cytometry ((apoptotic) cell death); means/SEM. **(a)** Significantly (*** P<0.0001; ** P = 0.0012) higher CLL cell death induction by the MCNA (highest efficacy for Huni132) than in PBMC; a less pronounced selectivity for bendamustine. Solvent associated cell death corresponded to spontaneous death rates. **(b)** CLL with low-risk karyotypes (normal or isolated del13q14; n = 12) vs 9 CLL carrying the high-risk aberrations del11q or del17p (including 4 clinically fludarabine refractory patients) show no significant differences in MCNA sensitivity. **(c)** Immunoblot-based detection of PARP cleavage induced by MCNA in 2 CLL representing the clinical/cytogenetic profile of investigated samples (β-actin corrected densitometric values). **(d)** Increased in vitro cell death upon combination of MCNA (5 μM) with 25 μM bendamustine (data from 5 CLL) including a synergism for Huni132 and bendamustine (dashed lines; * P = 0.006). Bottom: corresponding levels of cleaved PARP (immunoblot, β-actin corrected densitometric values) of 2 exemplary CLL. **(e)** CLL B-cells from clinically fludarabine refractory patients (n = 9, including the 4 from (b); cases #32-40 in Table S2) were cultured (48 h) in the presence of fludarabine (5 μM), bendamustine (25 μM), or the 2 most efficient MCNA (10 μM). Cell viability after bendamustine (*** P = 0.0003) and especially MCNA (**** P<0.0001) was significantly reduced while lost in vitro fludarabine sensitivity paralleled the clinical resistance. **(f)** MCNA, especially Huni132 (10 μM), more than bendamustine (25 μM), overcome the strong pro-survival support mediated to CLL cells by cocultures with NKtert BMSC. Immunoblots depict PARP cleavage in 2 representative cases (N = 5 total). Densitometric values (β-actin corrected) for cPARP/PARP are relative to those for Dmso (set as "1") for each of the two culture conditions. BMSC coculture reduces spontaneous (Dmso) PARP cleavage (densitometry: case 1 *5.4 (suspension culture) vs 0.4 (coculture), case 2 **0.67 vs 0.07). AnnexinV/7AAD-based cell survival (not shown) paralleling these data (suspension vs coculture) was: 70.3 % vs 83.3 % (case 1) and 71.4 % vs 84 % (case 2). Increasing TCL1 levels (β-actin corrected densitometric values; only Dmso in suspension culture set as “1”) also indicate an efficient protective feeder cell interaction [26]

Flow cytometry with AnnexinV (early-apoptotic surface expression of the neoepitope phosphatidyl-serine) combined with 7AAD or Hoechst stain (uptake upon late-apoptotic or necrosis-associated membrane disintegration) assayed the ability of our MCNA to trigger death of primary tumor cells from CLL patients and of healthy-donor derived PBMC. Initial estimations of effective compound dosages were performed by LD50 titrations (Additional file 1 Fig. S1). Next, already after 24 h all 4 tested MCNA, with the highest efficacy noted for Huni132, induced pronounced cell death in 25 CLL samples whereas normal PBMC were significantly less sensitive towards MCNA treatment (Fig. 2a). Solvent (Dmso) induced cell death was negligible (mean/SEM: PBMC 3.7 %/0.9 and CLL 5.4 %/1.3). This overall selectivity by the MCNA was greater than observed for bendamustine at this time point and for fludarabine, compared to which the MCNA also proved to be more B-cell specific (Fig. 2a, Additional file 1 Fig. S2).

To evaluate the activity of the MCNA across different risk subsets of CLL (Additional file 1 Tables S1, S2), we compared post-exposure cell survival of cytogenetically high-risk cases [5, 6, 24] carrying 11q or 17p deletions (N = 9,) with 12 good-prognosis CLL (normal karyotype or isolated 13q14 deletion). The MCNA triggered cell death was at comparable rates, independent of cytogenetic disease subset (Fig. 2b). Responses were also mostly irrespective of IGHV mutational status or of previous therapy (Additional file 1 Fig. S3). Furthermore, the MCNA-induced biochemical response of PARP cleavage, a distal event in several routes of programmed cell death, was similar between samples of different karyotypes and from patients with distinct disease stages (Fig. 2c).

Next, we evaluated potential cooperative effects of our novel substances with bendamustine, a well-established clinically active drug with a good therapeutic index (low toxicity) in CLL. We preferred this well-described alkylator for this and some subsequent sets of experiments over the conventional adenosine nucleoside fludarabine based on bendamustine’s faster reaction kinetics (Additional file 1 Fig. S1). The fludarabine metabolism with synthesis of cellular F-ara-ATP peaking after ≈ 48–72 h allows reliable efficacy recordings for fludarabine starting only at 48 h. Therefore, and since we already expected the MCNA not to act as “classical” nucleosides [23], bendamustine appeared as the better comparison when the 24 h MCNA effects were studied.

Freshly isolated primary leukemic cells from 5 CLL patients were treated for 24 h with 5 μM of each MCNA, alone or in combination with 25 μM bendamustine followed by AnnexinV/7AAD flow cytometry and immunoblot (PARP cleavage) analysis. There were at least additive effects across all combinations, with the most significant cooperation observed for bendamustine with Huni132 (P = 0.006) showing even a synergistic relationship (Huni132: 79.8 ± 9, Bendamustine 70.8 ± 9, Bendamustine + Huni132 46.4 ± 7.5; all Mean ± SEM; Fig. 2d). These effects were further corroborated by an increased cleavage of PARP seen for those drug combinations (Fig. 2d, bottom).

Fludarabine resistance in CLL remains a clinical problem. We therefore analyzed samples from 9 CLL patients with clinically fludarabine-refractory disease for responses to the MCNA; as 4 cases to all 4 MCNA (see cytogenetic high-risk subsets, above and Fig. 2b) or as all 9 cases to only Huni132 and Me-N69 (Fig. 2e). Importantly, the MCNA and in part also bendamustine were still efficient in the context of clinical and in vitro fludarabine resistance (Fig. 2e).

The high apoptotic threshold of CLL B-lymphocytes is significantly determined by milieu-derived signals. The direct contact between leukemic cells and stromal components, i.e. BMSC, was shown to protect from spontaneous and drug-induced apoptosis [25, 26]. We therefore explored the influence of the MCNA on CLL cell viability in a stromal cell coculture system as this represents a higher fidelity model of niche-mediated resistance than suspension cultures. For this, CLL cells were kept either in suspension or as cocultures with the human BMSC line NK-tert. After 24 h of drug exposure, cell death rates and levels of cleaved PARP were recorded. In line with our previous findings [26], we observed upregulation of protein levels of the pro-survival oncogene TCL1 in CLL cells upon NK-tert co-culture (Fig. 2f). The BMSC coculture protected CLL cells from spontaneous and in part from bendamustine-induced cPARP-induction/cell death. In contrast, the MCNA consistently evoked marked cell death also in the presence of BMSC-mediated pro-survival stimuli; in the case of Huni132 nearly to the same extent as in the suspension cultures. For cPARP densitometry see Fig. 2f; corresponding AnnexinV/7AAD double-negative fractions of surviving cells (not shown) were (suspension vs coculture; percentage-means ± SEM): Huni132: 23.3 ± 3.2 vs 34.6 ± 10.2, Huni218: 56.8 ± 8.2 vs 70.9 ± 7.6, Me-N69: 38.8 ± 7 vs 62 ± 7.2, Dia-Me-N69: 66.5 ± 8.2 vs 86 ± 4.9, and bendamustine: 89.3 ± 4.5 vs 94.5 ± 0.9. As the feeder cells themselves remained unaffected by the treatment with the drugs (Additional file 1 Fig. S4), the differential induction of leukemia cell apoptosis was not due to loss of BMSC protection, rather CLL-cell specific. Furthermore, only incubation of CLL cells in supra-physiological 100 % (instead of 10 %) serum of either fetal calf or human origin confered some degree of protection from Huni132 mediated cell death (Additional file 1 Fig.S5).

Taken together, we demonstrated thus far that our novel MCNA efficiently and specifically trigger cell death in CLL, irrespective of the disease risk group or the presence of protective cell-cell mediated micro-environmental signals.

### The cell death induced by organometallic nucleosides in CLL is non-necroptotic and non-autophagic as well as independent of caspase-3 and p53

Currently, at least 3 mayor pathways of programmed cell death are distinguished by characteristic cell fates, namely apoptosis, autophagy, and necroptosis [27–29]. Upon MCNA exposure, we identified morphologic changes that were rather characteristic for apoptosis, namely pyknotic nuclei and cytoplasmic condensations (not shown). They paralleled the pronounced AnnexinV induction (above). However, to discriminate in more detail among the pathways involved in the MCNA-mediated CLL cell death, we employed Z-VAD and necrostatin-1 as specific inhibitors of caspase-mediated apoptosis and caspase-independent necroptosis, respectively. In addition, we tracked the autophagy-specific proteolytic conversion of the marker protein LC3B-I to autophagosome associated LC3B-II. We also analyzed the cleavage of PARP, which is mainly, but not exclusively [30], triggered by the activation of the caspase signaling cascade.

**Fig. 3:**
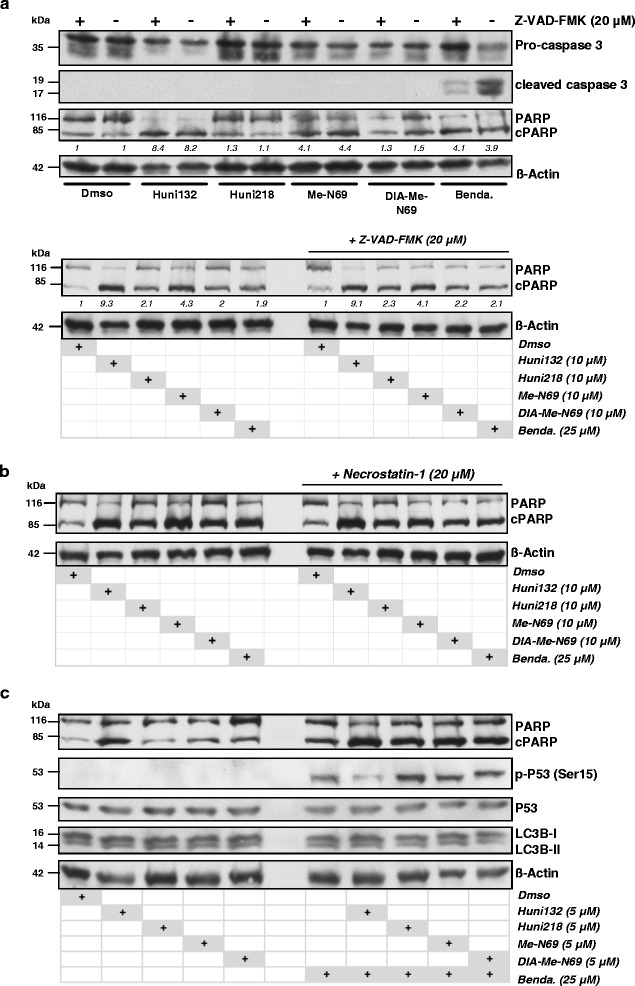
The cell death induced by organometallic nucleosides in CLL is non-necroptotic and non-autophagic as well as independent of caspase-3 and p53. Representative immunoblots (12 % SDS gels, 20 μg protein load) of independent experiments on 8 CLL patient samples. Suspension cultures were untreated or treated with 10 μM of each of the 4 metal-containing nucleoside analogues (MCNA) for 24 h following a 1 h pre-incubation with the “analytic” inhibitor. **(a)** The MCNA trigger cleavage of PARP at various degrees without induction of cleaved effector caspase-3 (top panel) and irrespective of the presence of the pan-caspase inhibitor Z-VAD (20 μM; top and bottom panel). In contrast, bendamustine (25 μM) mediates caspase-3 activation, which is Z-VAD responsive (top panel). **(b)** The inhibitor of necroptosis Necrostatin-1 does not noticeably affect the MCNA induced PARP cleavage in CLL cells (staurosporine control experiments in online Supplements). **(c)** PARP cleavage in CLL cells promoted by the MCNA is neither associated with pSer15-P53 activation nor with changes in levels of the autophagocytic marker protein LC3B-II (rapamycin control experiments in online Supplements). The combination of the MCNA with the alkylator bendamustine enhances PARP cleavage alongside a specific p53-phospho-activation, but without noticeable changes in levels of converted LC3B-II

We found that the MCNA lead to PARP cleavage in CLL cells without preceding caspase-3 activation (Fig. 3a). Neither Z-VAD nor necrostatin-1, were able to prevent the cleavage of PARP by the MCNA (Fig. 3a,b, Additional file 1 Fig. S6). However, the PARP-inhibitor olaparib mitigated MCNA-mediated CLL cell death (Additional file 1 Fig. S7). This suggested that the MCNA-mediated cell death is to a large degree PARP dependent, but not promoted through classical apoptosis or necroptosis. Although the presence of Z-VAD could not fully prevent the generation of cleaved PARP by bendamustine exposure, this pan-caspase inhibitor suppressed the otherwise robust induction of caspase-3 cleavage products by bendamustine. Furthermore, while bendamustine triggered strong P53 Ser15-phosphorylation, none of the MCNA evoked such p53 phospho-(p)-activation (Fig. 3c). PARP cleavage and higher cellular death rates by MCNA treatment were not associated with autophagic LC3B conversion (Fig. 3c), which however was the case for the positive control of rapamycin (Additional file 1 Fig. S8).

Together these data suggest that the programmed cell death mediated by the MCNA can be considered as a PARP mediated, caspase-3 independent, hence non-classical, way of apoptosis that does not involve P53. This distinguishes these substances strikingly from conventional agents like bendamustine, which acts at least to a relevant part caspase-3 dependent and via P53.

### The induction of CLL cell apoptosis by metal-containing nucleosides involves a selective ROS dependence

**Fig. 4:**
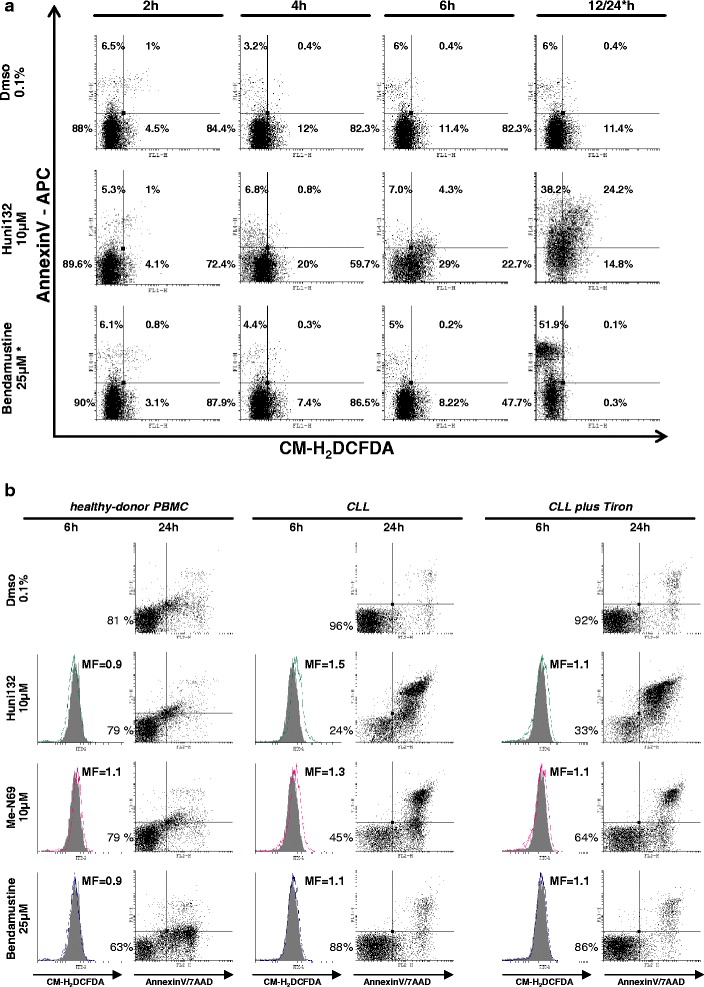
The induction of CLL cell apoptosis by metal-containing nucleosides is ROS dependent. Flow cytometry with representative dot plots of 3 CLL samples. **(a)** Increasing ROS levels (detected by CM-H2DCFDA probe) following exposure to metal-containing nucleosides (MCNA; example here Huni132) were noticeable at 4-6 h and were associated with the induction of early apoptosis (Annexin-V). Apoptosis induced by bendamustine was not paralleled by increases of cellular ROS. * For bendamustine data are shown at 24 h instead of 12 h due to different cell-death kinetics, however, at no time-point of bendamustine exposure a ROS increase was noted. **(b)** The increasing ROS levels (CM-H2DCFDA, solid grey histograms represent Dmso controls) at 6 h that precede the induction of cell death by the MCNA (x: AnnexinV, y: 7AAD; max. at 24 h), but not by bendamustine, are preferentially observed in CLL tumor cells as compared to healthy donor peripheral blood mononuclear cells (PBMC). Pre-incubation (1 h) with several ROS scavengers, here Tiron (10 mM), partially abrogated the MCNA mediated ROS increase and reduced the extent of overall apoptosis (AnnexinV+ fraction). MF: mean fold changes in mean fluorescence intensity. Lower-left quadrant %-ages indicate AnnexinV/7AAD double-negative viable cells

Caspase-independent cell death is important for organismal protection upon failure of the caspase-mediated pathways, and can be triggered in response to cytotoxic agents [12, 23, 31]. Damage of organelles including mitochondria and increases of cellular ROS levels are frequently involved in such caspase-independent cell death [12]. Based on their chemical properties (ionic metals) we predicted our MCNA to induce ROS in a Fenton’s reaction (iron-catalyzed hydrogen peroxide synthesis). Therefore, we analyzed cellular levels of ROS in MCNA-treated CLL (n = 3) cells in relation to induced apoptosis to test for this possible caspase-independent mode of MCNA action. Recordings of cellular retention of the fluorescent (oxidized) CM-H2DCFDA dye measured ROS levels at different time points after drug exposure (2 h, 4 h, 6 h, and 12/24 h). Increased ROS levels were revealed already at 4–6 h after MCNA exposure (Huni132, Fig. 4a), while bendamustine-treated cells showed unchanged ROS levels. The earliest AnnexinV expression, however, was detectable after 12/24 h of drug treatment demonstrating that the ROS increase precedes the induction of apoptosis. According to these data we speculated that increasing ROS levels are causally linked to the induction of early apoptosis by the MCNA, which stands in contrast to the p53-mediated activation of a ROS-independent DNA-damage stress response and caspase-mediated apoptosis by bendamustine.

To highlight the functional and specific role of ROS in MCNA mediated killing of leukemic cells, we investigated cell death (3 CLL cases) in the presence of the cell-permeable ROS scavengers Tiron (chelator, scavenger of superoxide ions and free electrons [32]), NAC (multimodal; antioxidant through GSH synthesis [33]), and Deferasirox (oral iron-chelator in clinical use). These substances all mitigated the redox-induced apoptosis by the MCNA as we observed a subtotal reduction of ROS and a reduced degree of MCNA-triggered cell death in their presence (Tiron, Fig. 4b). From that, we conclude that the mode of anti-leukemic action of the MCNA is at least to a high degree, but possibly not entirely, ROS dependent. In agreement with our data on a rather selective cell death induction by MCNA treatment in CLL tumor cells over healthy-donor PBMC (Fig. 2a), we were also not able to detect MCNA-induced ROS increases preceding the low degree of apoptosis in these normal cells (Fig. 4b). This could provide a rationale for a low cytotoxicity of MCNA in healthy tissues.

We proposed that a major underlying factor for the tumor-specific activity of the MCNA is the disturbed redox equilibrium present in CLL cells [8, 10, 34]. In the search for causative factors of elevated ROS in CLL [8], we postulated that besides an increased mitochondrial mass [34], oncogenic stress represents a leading force towards this part of the aberrant redox signature of CLL. Therefore, we decided to investigate this potential vulnerability aspect further by following the hypothesis that one of the central oncogenes in CLL, TCL1, might represent a causal link. This is reasoned by TCL1’s activating interaction with AKT [19, 35], a central growth-promoting signaling node and an established inducer of oxygen free radicals [36].

### Higher levels of ROS and an associated increased sensitivity towards metal-containing nucleosides are linked to high TCL1 oncogene expression

**Fig. 5:**
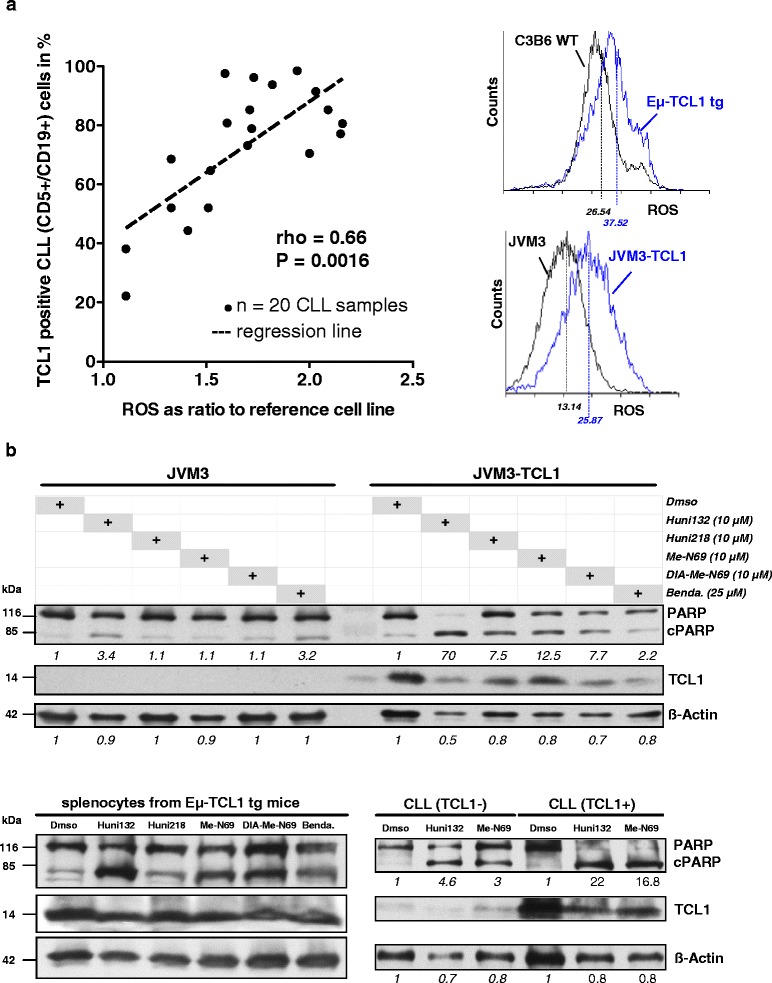
Higher levels of ROS and an increased sensitivity towards metal-containing nucleosides are associated with high TCL1 oncogene expression. **(a)** Left: overall cellular ROS levels (CM-H2DCFDA flow cytometry) positively correlate with the percentage of TCL1-expressing tumor cells in 20 freshly isolated CLL (Spearman’s rho = 0.66, P = 0.0016). Adjustments by DoHH2 control cells in each individual measurement to address inter-experimental variations are outlined in Materials and Methods. Right-top: basal cellular ROS levels in splenic B-cells from 7 months old TCL1-transgenic mice (Eμ-TCL1 tg) are higher than in those from age-matched animals of identical C3HxC57BL/6 (C3B6) wild-type (WT) background (representative histogram with mean fluorescence intensities (MFI) from measurements on 3 different mice). Right-bottom: Introduction of TCL1 into JCM3 CLL-like cells (stable transfectants JVM3-TCL1) leads to higher ROS levels (MFI; representative example of 3 independent measurements). **(b)** Top: representative Western blot of 3 experiments of 24 h of metal-containing nucleoside treatment of JVM3 cells. A stronger PARP cleavage is observed in the TCL1-expressing sub-line. The opposite is noted for the ROS-independent alkylator bendamustine. Numbers represent densitometric ratios to Dmso controls of cleaved/non-cleaved PARP normalized to ß-Actin loading. Bottom-left: the same pattern of differential induction of PARP cleavage across the substances is seen in leukemic Eμ-TCL1-tg mice (24 h, 1 representative of 3). Bottom-right: immunoblot showing PARP activation in a selected pair of 6 CLL patient samples demonstrating a stronger PARP cleavage in TCL1-high cases than observed for tumors expressing this oncogene at hardly detectable levels (Huni132 and Me-N69, both at 10 μM)

Apart from their function in programmed cell death, intracellular ROS are known to regulate a number of tumor-associated signaling cascades particularly PI3K/AKT and NFkB, the 2 major effector pathways of the lymphoid oncogene TCL1 [35, 37, 38]. To explore the potentially tight relation of tumor cell inherent ROS to TCL1, first the number of TCL1+ cells within the CD5+/CD19+ CLL tumor cell population was correlated with the overall leukemic ROS levels by flow cytometry. In PB samples from 20 CLL, we observed a significant association (rho 0.66, P < 0.002) of ROS levels with the percentage of TCL1+ cell within the malignant clone (Fig. 5a left). These results were confirmed in systems of modulated TCL1. Eμ-TCL1-tg mice develop noticeable CD5+ B-cell proliferations at 6–10 months resulting in overt CLL after 12–15 months [18]. Fittingly, the ROS levels in splenocytes from 7-months old pre-leukemic, (Fig. 5a right-top) and leukemic (not shown) TCL1-tg mice were significantly higher than in splenocytes from age-matched C3HxC57BL/6 wild-type controls. Similarly, introduction of TCL1 in the CLL-like cell line JVM3 led to strongly elevated ROS levels as compared to the parental cells (Fig. 5a right-bottom). The findings from both experimental systems argue against the possibility that the elevated ROS levels are predominantly a general cancer phenomenon unrelated to TCL1 (TCL1 transfection of an already fully transformed cell line; analysis of pre-leukemic mice) or to be seen in the context of organismal/lymphocyte ageing (age-matched animals). More likely, the results suggest that the elevated TCL1 expression, characteristic for CLL [19, 20], stands in a causal relation to a dysbalanced redox state resulting in aberrantly high ROS levels.

We next analyzed whether TCL1 overexpression, linked through the elevated ROS levels translates into altered sensitivity towards our MCNA. Indeed, upon MCNA treatment the amount of cleaved PARP was noticeably higher in the TCL1-carrying JVM3 CLL-like B-cells than in the parental line, which was confirmed by the number of AnnexinV expressing cells (not shown). In contrast, bendamustine sensitivity was slightly reduced by stable TCL1 transfection (Fig. 5b, top), a phenomenon we observe more strikingly for fludarabine in vitro and in the clinical setting [19]. Similarly, TCL1-driven murine leukemias (Eμ-TCL1-tg splenocytes) showed a good response to our novel agents (Fig. 5b bottom-left). Furthermore, MCNA treatment elicited much stronger cytotoxic responses in TCL1-positive CLL as compared to TCL1-negative cases (Fig. 5b bottom–right). Together, these data demonstrate that high TCL1 oncogene expression directly associates with elevated cellular ROS levels and by that mediates an increased vulnerability towards MCNA.

### Dependence on mitochondrial energetic flux renders TCL1-positive cells more susceptible towards agents interfering with mitochondrial homeostasis

**Fig. 6:**
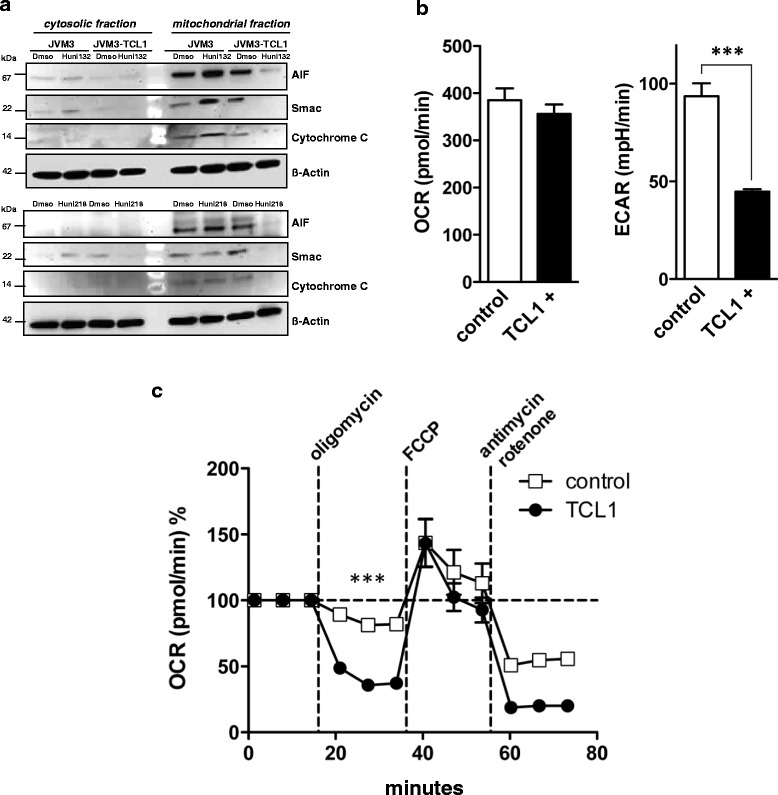
Dependence on mitochondrial energetic flux renders TCL1-positive leukemic cells more susceptible towards agents interfering with mitochondrial homeostasis. **(a)** Representative immunoblots on cytosolic and nuclear fractions from 3 experiments on CLL-like JVM3 cells and their TCL1-positive sub-line after 24 h of metal-containing nucleoside treatment. Treatment with these substances reduces the levels of the mitochondrial proteins AIF, smac, and cytochrome c in this subcellular fraction, specifically in the TCL1-overexpressing sub-line. **(b, c)** Mitochondrial respiration (oxygen consumption rate; OCR) and extracellular acidification rate (ECAR; the result of anaerobic glycolysis) were measured (XFe96 flux analyzer) under basal conditions and in response to the indicated substances in JVM3 cells (control) and those transfected with TCL1. **(b)** Left: baseline OCR is similar between JVM3 cells and their stable TCL1 transfectants, while a lower degree of ECAR in the presence of overexpressed TCL1 is noted. **(c)** The effects on OCR are shown as percentages of baseline (set as 100 %) for each treatment (substances added after 3^rd^, 6^th^, and 9^th^ time-point). The stronger changes in OCR after oligomycin (ATP synthase inhibitor to differentiate the ATP-linked respiration from the proton leak) in the TCL1-positive sub-line are indicative of higher degrees of respiration (OCR) linked to ATP synthesis. Maximal respiratory capacity as determined by addition of the uncoupling agent FCCP was not different. Antimycin A/rotenone stop the reaction (respiration) and the remaining level of OCR (non-mitochondrial respiration) is lower in the TCL1-carrying sub-line. All experiments of (b)-(c) were performed at least 6 times with *** indicating P < 0.001 and bars for the standard error of the mean

The sensitivity to our MCNA that TCL1 oncogene overexpression conferred via elevated ROS was intriguing as it stands in contrast to a general TCL1-associated resistance towards classical apoptosis inducing agents [19]. Therefore, we asked for the specific underlying mechanisms of this vulnerability. Thus far, our results showed that the apoptosis induced by MCNA does not rely on active caspase-3 (Fig. 3a). We next investigated the release of mitochondrial proteins in response to apoptotic stimuli with particular focus on the flavoprotein apoptosis-inducing factor (AIF), a known mediator of caspase-independent apoptosis [31], Smac, the suppressor of the inhibitors of apoptosis proteins (IAP) [39], and cytochrome c, an electron transport protein released from mitochondria during the early stages of apoptosis [40]. JVM3 CLL-like cells and their stable TCL1 transfectants were treated with Huni132 or Huni218, and the protein levels of AIF, Smac, and cytochrome c were analyzed in cytosolic and mitochondrial fractions by immunoblots. There were no obvious uniform changes in AIF, Smac, or cytochrome-c levels at baseline (untreated cells). However, most consistently, exposure to the MCNA resulted in mitochondrial depletion of all three proteins in the TCL1-transfected JVM3-cells (Fig. 6a). A simultaneous cytosolic accumulation of these released molecules was not captured at the parallel time points, which points to different kinetics of their degradation and established nuclear trans-localization. Overall, these data implicate that the presence of TCL1 promotes the apoptotic effects of the MCNA at the mitochondrial level and involves mediators of caspase-independent pathways.

We next monitored bio-energetic rates of the two major sources of cellular ATP generation, glycolysis and mitochondrial respiration, in the context of substance challenge and in relationship to the impact of TCL1. Interestingly, basal mitochondrial respiration (OCR) was not increased in TCL1-transfected cells (Fig. 6b). However, basal aerobic glycolysis as indicated by lactate production (ECAR), was significantly lower in the JVM3-TCL1 cells (Fig. 6b). Furthermore, treatment with the complex-V inhibitor oligomycin reduced the OCR on average to 41 ± 7 % (of baseline level) in the TCL1-transfected JVM3 cells vs 84 ± 4 % in the TCL1-negative JVM3 control (Fig. 6c). Since the mitochondrial complex-V (F0F1-ATPase) couples the electron chain to ATP synthesis, this result of a higher oligomycin-sensitive fraction indicates that TCL1 abundance is associated with a significantly higher fraction of oxygen consumption being coupled to ATP-synthesis (higher ATP turn-over). The maximal respiratory capacity as determined by addition of the uncoupling agent FCCP was not impacted in TCL1’s presence. The antimycin/rotenone insensitive OCR fraction was lower in the TCL1 condition indicating a lower contribution of the non-mitochondrial component of OCR to overall respiration under TCL1 impact.

In conjunction, these data implicate a TCL1-mediated shift towards a higher relative contribution of the mitochondrial metabolism over glycolysis to overall ATP generation and over non-mitochondrial sources of oxygen consumption to global respiration. It could partially provide an explanation for the increased ROS levels mediated by TCL1 (Fig. 5). At the same time a stronger dependence on mitochondrial energetic flux seems to render TCL1+ cells more susceptible towards agents interfering with mitochondrial homeostasis such as the tested MCNA.

### The novel metal-containing nucleosides induce a rapid mitochondrial depolarization in primary CLL cells

**Fig. 7:**
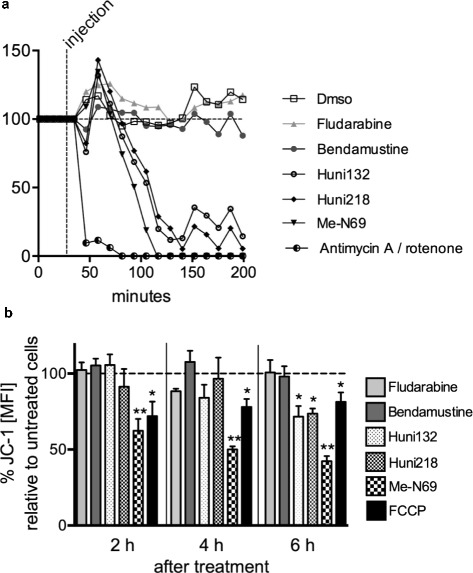
The organometallic nucleosides induce a rapid mitochondrial depolarization in primary CLL cells. **(a)** Respiration (oxygen consumption rate; OCR) is measured in primary CLL cells (n = 3) under basal conditions and in response to 10 μM fludarabine, 25 μM bendamustine, and 10 μM of the 3 most active metal containing nucleoside analogues (MCNA) in addition to a condition of 3 μM of the electron transport chain inhibitors antimycin A/rotenone (specifically block mitochondrial respiration). OCR quickly and drastically drops after exposure to the MCNA, but not after bendamustine or fludarabine. Percentages of OCR as compared to the baseline measurement (“before injection”, set as 100 %) are shown. **(b)** The mitochondrial electrochemical membrane potential (∆ΨM) was semi-quantified in primary CLL-cells (n = 3) by flow cytometry using the potentiometric dye JC-1. Histograms of a representative donor are shown in Additional file 1 Fig. S9. Changes in JC-1 mean fluorescence index (MFI) were assessed upon treatment with 10 μM fludarabine, or 25 μM bendamustine, or 10 μM of each of the 3 most active MCNA. FCCP (1.5 μM) an ionophore uncoupler was used as a positive control for reducing the mitochondrial membrane potential and accordingly JC-1 staining. The MCNA, but neither fludarabine nor bendamustine, induce marked decreases in ∆ΨM. Summarized results represent percentages of the MFI of untreated control cells (set as 100 %). Significance levels: *P ≤ 0.05; ** P = 0.002

Under physiologic conditions, production of ROS is a byproduct of normal ATP-dependent mitochondrial respiration [34]. Given the selective efficacy of our MCNA in CLL acting through ROS induction and based on the modulatory impact of the CLL oncogene TCL1 on mitochondrial metabolism, we hypothesized that the MCNA induce cell death in primary CLL cells by significantly affecting their mitochondrial function. We analyzed the respiration and membrane potential of mitochondria in CLL samples after incubation with MCNA. We found that mitochondrial respiration (OCR) is significantly reduced upon MCNA exposure compared to bendamustine-, fludarabine-treated or untreated CLL samples (Fig. 7a). In addition, we assessed the mitochondrial membrane potential (∆ΨM) as an established marker for mitochondrial fitness. The energy produced during mitochondrial respiration is maintained as a high ∆ΨM whereas declining mitochondrial function or their destruction is associated with a ∆ΨM loss. As anticipated, application of the MCNA, but neither bendamustine nor fludarabine, resulted in a rapid mitochondrial depolarization in primary CLL cells suggesting that mechanistically our MCNA promote ROS production in tumor cells by affecting mitochondrial respiration and membrane polarization (Fig. 7b, Additional file 1 Fig. S9).

## Discussion

Cancer cells undergo various adaptations to cope with (oncogenic) stresses and to secure a high-level energy supply [13]. Targeting these metabolic consequences of oncogene impact or loss of safeguarding tumor suppressor function is increasingly recognized as a more fundamental and specific approach than attempting to intercept in distinct oncogene addictions [36]. Oxidative stress, as exerted by ROS, is strongly implicated in malignant transformation and in responses to therapeutic agents. ROS act pro-tumorigenic as signaling intermediates, e.g. in CLL downstream of BCR signals [14, 15], or by their DNA-mutagenic effect. Although the dysbalanced redox homeostasis of the neoplastic phenotype is associated with elevated ROS levels, cancer cells also have a high anti-oxidant capacity to ensure compatible ROS levels. For CLL cells, we showed earlier that such a stress adaptation also involves an enhanced mitochondrial biogenesis [34]. Overall, there is a high level of oxidative stress as well as an elevated response to it in cancer, hence, both sides of a delicate ROS inducer/scavenger equilibrium represent promising Achilles’ heels for intervention [10, 36].

We previously exploited this principle by organochalcogens acting as sensor/effector catalysts inducing intolerably high ROS levels especially in redox burdened cancer cells, including those of CLL [7, 8]. Here, we expanded on similar promising proof-of-principle data of another rare set of substances, namely organometallic nucleoside analogues [21–23]. We had identified before the crucial roles of their cytosine nucleobase and metal core (here now ferrocene, ruthenocene, and Fe(CO)_3_) in cell death induction in lymphoblastic leukemia [21]. We additionally provided the compounds with a protecting 5**’**-CH2O-TDS(thexyldimethylsilyl) substituent, because this promised to add cytotoxic potential compared to a sole 5**’**-OH group [41] in our lymphoblast systems. [21] In the present study, the combination of these 3 ‘active’ groups in the 4 selected MCNA conferred a high in vitro cytotoxic efficacy in CLL cells. This was specific over normal hematopoetic cells and could overcome the protective effect by modeled milieu-derived signals (BMSC cocultures). Most importantly, the MCNA were equally efficient in CLL carrying low-risk aberrations vs those with–11q or/and–17p, which are known to confer resistance to conventional cytostatics, such as fludarabine or bendamustine [24, 42]. In fact, our MCNA induced impressive rates of cell death in the 9 clinically fludarabine-refractory cases, of which at least 5 carried a–11q or/and–17p high-risk lesion. Another poor-risk determinant, unmutated (U) IGHV status, did not predict a poorer MCNA response. In fact there was a tendency towards a higher efficacy of certain MCNA in U-CLL, which might find its correlate in their generally higher TCL1 levels [20] in association with higher ROS levels (below).

These highly desirable features of targeting niche-protected cells or those of genetically or clinically defined resistance to agents inducing classical apoptosis challenged us to investigate the MCNA-mediated modes of cell killing in more detail. We discovered here an induction of non-autophagic, non-necrotic apoptotic cell death by MCNA exposure that did not entail p53 activation and effector caspases. Moreover, this unconventional MCNA-evoked apoptosis involved early cellular increases of ROS, which proved indispensible for the induction of cell death. These characteristics also distinguished our MCNA from traditional cytostatics like bendamustine. It makes their mechanistic action an attractive principle to overcome more efficiently the resistance to classical apoptosis inherent to CLL cells.

Generation of ROS, containment of their cytotoxic potential via compartmental sequestration, and release of caspase-dependent or -independent (e.g. AIF) executioners of apoptosis are mediated mainly by mitochondria. Earlier, we described that non-mitochondrial production of ROS by the membrane-bound NADPH-oxidase (NOX) does not contribute to the elevated ROS levels in CLL [34]. Fittingly, we found here that the increases in cellular ROS by our MCNA preceding the induction of CLL cell death were associated with severe disturbances of mitochondrial function. Both, mitochondrial respiration (oxygen consumption) and membrane potential were markedly reduced upon MCNA treatment. Surprisingly, bio-energetic characterizations of CLL cells are still rare. However, this study corroborates previous notions by us [7, 8, 34] and others [9, 10, 12, 43] that the amplifying interconnection of elevated ROS generation and high-level activity of adaptive mechanisms represent a metabolic profile of CLL to be exploited more intensely in the future.

As we showed before that the mitochondrial electron transport chain (mETC) in CLL is not uncoupled [34], the actual causes of mitochondrial ROS accumulation in CLL cells remain one of the most central questions. In fact, we demonstrate for the first time data that implicate the tumorigenic adapter molecule TCL1, an established signaling modulator in CLL pathogenesis, in driving elevated ROS levels. As underlying, we observed a reduction of aerobic glycolysis and a higher fraction of oxygen consumption coupled to ATP-synthesis, both to be mediated by TCL1. This strongly suggests that TCL1 renders cells more dependent on mitochondrial energetic flux through which it acts as a powerful promoter of intrinsic ROS overproduction in CLL.

TCL1 stands in multiple functional relationships to other redox regulatory molecules of relevance in CLL. For example, the TCL1-activated target kinase AKT increases ROS by a fueled oxidative metabolism. Its activation can also mediate selective pressure towards a more ROS tolerant phenotype as it can sensitize cells to oxidative stress owing to the inactivation of Foxo transcription factors, which in turn reduces expression of anti-oxidant enzymes [44]. Another TCL1-cooperating factor ATM [45], can be activated by ROS in the absence of DNA double-strand breaks. Its safeguarding program of ROS sensing and protective induction of autophagy [46] likely fails in the context of genetic ATM deficiency as found in up to 20 % of CLL, which are the cases with the highest TCL1 levels [19, 20].

To build therapeutic principles around the non-kinase chaperone TCL1 is a challenging task [47]. Therefore, it is most intriguing that we discover here how TCL1 confers a specific therapeutic susceptibility towards substances interfering in mitochondrial homeostasis, particularly our MCNA. We describe a TCL1-mediated enhanced dependence on mitochondrial respiration in association with elevated ROS. Furthermore, in the context of MCNA exposure TCL1 promoted a marked mitochondrial depletion of in part caspase-independent apoptotic factors, such as AIF. Although CLL is a mostly TCL1-overexpressing disease, dysregulated TCL1 is also found in other B-cell lymphomas [48], T-cell prolymphocytic leukemia [49], and some solid tumors [50]. Therefore, it will be of interest to test if its disease-promoting properties can be turned into an exploitable vulnerability in a broader spectrum of entities by drugs that target redox systems or the mitochondrial metabolism. At the very least, we established here that the high-level expression of otherwise pro-survival TCL1 marks the problematic aggressive CLL subset with resistance to conventional treatment [19, 20], but with elevated sensitivity to redox-based strategies [43].

## Conclusions

The aberrant phenotype of high-level oxidative stress and altered mitochondrial metabolism in CLL can be explained by the impact of the lymphoid oncogene TCL1. Redox-active substances such as organometallic (non-conventional) nucleosides confer specific cytotoxicity to such TCL1-overexpressing and ROS-burdened cells. With respect to the chemistry-translated detailed modes of action of our novel MCNA, we postulate also other mechanisms besides a sole redox modulating (ROS quantity and ROS composition) activity; the latter likely deriving from the metal unit. These can involve engagements of certain protein or receptor targets (predisposing steric bulk) or in the case of Me-N69, release of the signaling intermediate carbon monoxide (CO). Nevertheless, the clinical significance of their mode of action via p53- and caspase-independent induction of PARP-mediated non-classical apoptosis lies in the potential of such (redox-based) strategies to particularly overcome the notorious resistance of clonal subpopulations or defined disease subsets and to turn this into a marked interventional vulnerability. These data justify the next level of studies on the bioavailability and in vivo performance of (optimized) MCNA substances.

## Material and methods

### *CLL patients*

After obtaining written informed consent according to guidelines of the Declaration of Helsinki and institutional review board–approved protocols (#11–319) at Cologne University, 40 individuals with the diagnosis of CLL based on iwCLL criteria [51] provided peripheral blood (PB) samples, which were taken ≥1 month after any anti-CLL therapy. Their characteristics are indicated in Additional file 1 Tables S1 and S2.

#### Sample purification and cell culture

Primary human CLL B-cells and murine splenocytes were purified and cultured as described [52]. The minimum purity (CD19+ or CD5 + CD19+ B-cells) of samples from Eμ-TCL1 transgenic (tg) [18] mice and CLL patients was 90 %. Healthy-donor PB mononuclear cells (MC) were isolated by density-gradient centrifugation. Human and murine CLL cells were exposed to the substances (below). For CLL-feeder cell cocultures, 1 × 10E6 cells of the human bone marrow stroma cell (BMSC) line NK-tert reached ≈ 60–70 % non-confluent density 24 h after plating, after which freshly isolated primary CLL cells were added [26]. Drug exposure for 24 h started after most CLL cells had firmly attached to the BMSC feeder layer (4 h) [26]. DoHH2 B-cell lymphoma and JVM3 CLL-like cells [53] as well as their TCL1-overexpressing sub-lines (stably transfected by a TCL1-overexpression construct [19, 54]) were cultured in RPMI1640 medium (Gibco, Life Technologies; Carlsbad, California), supplemented with penicillin (100 μg/ml), streptomycin (100 μg/ml), and 15 % fetal calf serum (FCS).

#### Compounds

All 4 organometallic nucleoside analogues used here were checked for purity by 1H nuclear magnetic resonance spectroscopy (NMR) after synthesis. The dry solids were dissolved in dimethylsulfoxide (Dmso) as stock solutions, which were kept at -20 °C with minimal exposure to light and used at indicated concentrations and exposure times (usually 10 μM for 24 h). Bendamustine (Mundipharma, Limburg, Germany) was prepared as a water-diluted stock solution and used at 25 μM. Rapamycin (20 μM) was from Cell Signaling Technology (Cambridge, UK). Olaparib (3 μM) and Staurosporine (1 μM) were from Selleckchem (Houston, Texas, USA). Tiron (4, 5-Dihydroxy-1, 3-benzenedisulfonic acid), N-acetylcysteine (NAC), Necrostatin-1 (5-(1H–Indol–3–ylmethyl)–3–methyl–2–thioxo–4–Imidazolidinone), and Dmso were from Sigma-Aldrich, Missouri, USA. Z-VAD-FMK (carbobenzoxy-valyl-alanyl-aspartyl-(O–methyl)- fluoromethylketone) was both from Promega (Fitchburg, Wisconsin, USA). Deferasirox ([4–[(3Z,5E)–3, 5–bis(6–oxo–1–cyclohexa–2, 4–dienylidene)–1,2,4–triazolidin–1–yl]benzoic acid) was from Novartis, Basel, Switzerland. The ROS-scavengers Tiron, Deferasirox, and NAC (all dissolved in water) were added to the cell cultures at 10 mM, 1 h before exposure to the organometallic nucleoside analogues. The necroptosis inhibitor Necrostatin-1 (20 μM), the pan-caspase inhibitor Z-VAD-FMK (20 μM), and the PARP inhibitor olaparib (3 μM) were added to the cultures simultaneously to the nucleoside analogues.

#### Analysis of apoptosis/cell viability

Dead cells or dying cells were quantified by flow cytometry (Gallios, Beckmann Coulter, Germany) via differential AnnexinV-expression alongside incorporation of 7AAD or Hoechst stain (Apoptosis Detection Kit I, BD Bioscience; New Jersey, USA); e.g. apoptosis rate: %-AnnexinV/7AAD or %-AnnexinV/Hoechst double-positive fraction. Differentiation between B- and T-cells was achieved by staining for CD19 Pacific Blue and CD3 APC (BioLegend; San Diego, USA). As generally observed [25], incubation of CLL cells in fresh medium for 24 h/48 h resulted in 5–30 % ‘spontaneous’ cell death. Therefore, in all experiments, untreated samples were collected at each time point and the values for drug-induced cell death were normalized to these controls. The colorimetric MTT (3–(4, 5–dimethylthiazol–2–yl)–2, 5–diphenyltetra-zolium bromide) or MTS (3–(4, 5–dimethylthiazol–2–yl)–5–(3–carboxymethoxyphenyl)–2–(4–sulfophenyl) 2H-tetrazolium inner salt) assays (both Promega; Fitchburg, Wisconsin, USA) assessed metabolic activity and by that cell viability (in triplicates per sample), used according to the manufacturer’s instruction.

#### Immunoblotting

Protein lysates were separated by SDS-PAGE, transferred, and probed with primary and secondary antibodies as described [19]. For the analysis of cytochrome-c and AIF, cytosolic and mitochondrial sub-cellular fractions were separated using the mitochondria isolation Kit for Cultured Cells (Thermo Scientific, Rockford, IL, USA). To ensure equivalent protein loading and transfer, all blots were probed for β-actin. Unless otherwise indicated, all Western blots shown in the figures are representative of three independent experiments. Membrane probing included the following primary antibodies: anti–human TCL1 (clone 1–21) [19]; anti-BCL2 (C-2), anti-AIF (D-20), and anti-β-actin (C-11) from Santa Cruz Biotechnology, Texas, USA; anti-phospho (p)-P53 (Ser15), anti-P53 (1C12), anti-caspase-3, anti-cytochrome c, and anti-PARP from Cell Signaling Technology, Massachusetts, USA.; anti-Smac/Diablo from BD Transduction Laboratories, Germany; and anti-human LC3B from Novus Biologicals, Colorado, USA. The HRP-conjugated species-specific secondary antibodies were from Dianova, Germany. Band detection by chemiluminescence (Amersham Buchler, Germany) was followed by densitometry by ImageJ (rsb.info.nih.gov/ij), including normalization to β-actin expression.

#### Flow-cytometric detection of ROS

Cellular ROS levels were determined according to the manufacturer’s instructions (ROS Detection Reagents kit, Invitrogen Life Technologies, Carlsbad, USA) by flow-cytometric recordings (Gallios, Beckmann Coulter, Germany) of CM-H2DCFDA fluorescence. To ensure consistency, we stained DoHH2-TCL1 cells (grown under constant conditions) in each experiment in parallel and corrected thereby for variable parameters/settings.

#### Extracellular flux assays

Bioenergetics of CLL-cells, B-cells, and JVM3-cells were determined using the XF96e Extracellular Flux Analyzer (Seahorse Bioscience, North Billerica, MA, USA). Cells were seeded in specialized tissue culture plates at an optimized concentration of 240.000 cells/well and subsequently immobilized using CELL-TAK (BD Biosciences). One hour prior to the measurement cells were incubated at 37 °C in a CO_2_-free atmosphere. First, basal oxygen consumption rate (OCR; indicator of mitochondrial respiration) and extracellular acidification rate (ECAR; indicator of lactic acid production or glycolysis) were recorded. Next, OCR and ECAR changes upon application of oligomycin (1 μM), the uncoupler FCCP (2.5 μM), a combination of antimycin (3 μM) and rotenone (3 μM) (XF Cell Mito Stress Test Kit, Seahorse Bioscience) as well as bendamustine (25 μM), and the metal-containing nucleosides (10 μM) were evaluated. All experiments were performed at least 6 times.

#### Mitochondrial membrane potential

Mitochondrial electrochemical membrane potential (∆ΨM) was assessed using the potentiometric dye JC-1 (JC-1 Mitochondrial Assay Kit, Cayman Chemical). Cells were stained with JC-1 according to the manufacturer’s instructions for 15 min at 37 °C followed by flow cytometry.

#### Statistics

Graphs were generated using Prizm (GraphPad, San Diego, CA). Data were expressed as means ± 95 % confidence interval (CI). Unpaired t-tests were used to compare the mean values of different experimental groups. Histograms from flow cytometry were obtained and analyzed using the Cyflogic software (http://www.cyflogic.com). Correlations between ROS levels and percentage of TCL1 positive CLL cells (CD5+/CD19+) were analyzed using Spearman correlation analysis. P values of <0.05 were considered statistically significant. LD50s (induced cell death in 50 % of treated cells) were calculated from the dose response curves via non-linear regression analysis in GraphPad Prism [54].

## Additional file

Additional file 1: Figure S1. Determination of substance LD50’s in suspension cultures of primary samples of chronic lymphocytic leukemia (CLL). **Figure S2.** The B-cell selective cytotoxic profile of the investigated MCNA in comparison to the more T-cell toxic fludarabine is particularly apparent in CLL. **Figure S3.** The novel organometallic nucleoside analogues induce marked in-vitro CLL cell death mostly independent of somatic immunoglobulin gene mutation rates and previous therapy. **Figure S4.** The MCNA do not compromise NK-tert stromal cell viability. **Figure S5.** Serum components influence MCNA-mediated cytotoxicity of CLL cells in vitro. **Figure S6.** The MCNA induce non-necroptotic CLL cell death. **Figure S7.** Diminished MCNA cytotoxicity through PARP inhibition in CLL cells. **Figure S8.** The programmed cell death induced by organometallic nucleosides is non-autophagic. **Figure S9.** Rapid mitochondrial depolarization in primary CLL-cells. **Table S1.** Patient demographics and baseline characteristics (summary). **Table S2.** Patient demographics and baseline characteristics (by case).
