# Supplementary material for: Organometallic nucleosides induce non-classical leukemic cell death that is mitochondrial-ROS dependent and facilitated by TCL1-oncogene burden
Source: Mol Cancer. 2015 Jun 4;14:114. doi: 10.1186/s12943-015-0378-1 (PMC4453051; doi:10.1186/s12943-015-0378-1)
Supplement: Additional file 1: Figure S1. — Determination of substance LD50’s in suspension cultures of primary samples of chronic lymphocytic leukemia (CLL). Figure S2. The B-cell selective cytotoxic profile of the investigated MCNA in comparison to the more T-cell toxic fludarabine is particularly apparent in CLL. Figure S3. The novel organometallic nucleoside analogues induce marked in-vitro CLL cell death mostly independent of somatic immunoglobulin gene mutation rates and previous therapy. Figure S4. The MCNA do not compromise NK-tert stromal cell viability. Figure S5. Serum components influence MCNA-mediated cytotoxicity of CLL cells in vitro. Figure S6. The MCNA induce non-necroptotic CLL cell death. Figure S7. Diminished MCNA cytotoxicity through PARP inhibition in CLL cells. Figure S8. The programmed cell death induced by organometallic nucleosides is non-autophagic. Figure S9. Rapid mitochondrial depolarization in primary CLL-cells. Table S1. Patient demographics and baseline characteristics (summary). Table S2. Patient demographics and baseline characteristics (by case). [file 12943_2015_378_MOESM1_ESM.docx]

**Supplemental data**

**Figure S1: Determination of substance LD50’s in suspension cultures of primary samples of chronic lymphocytic leukemia (CLL).**

**
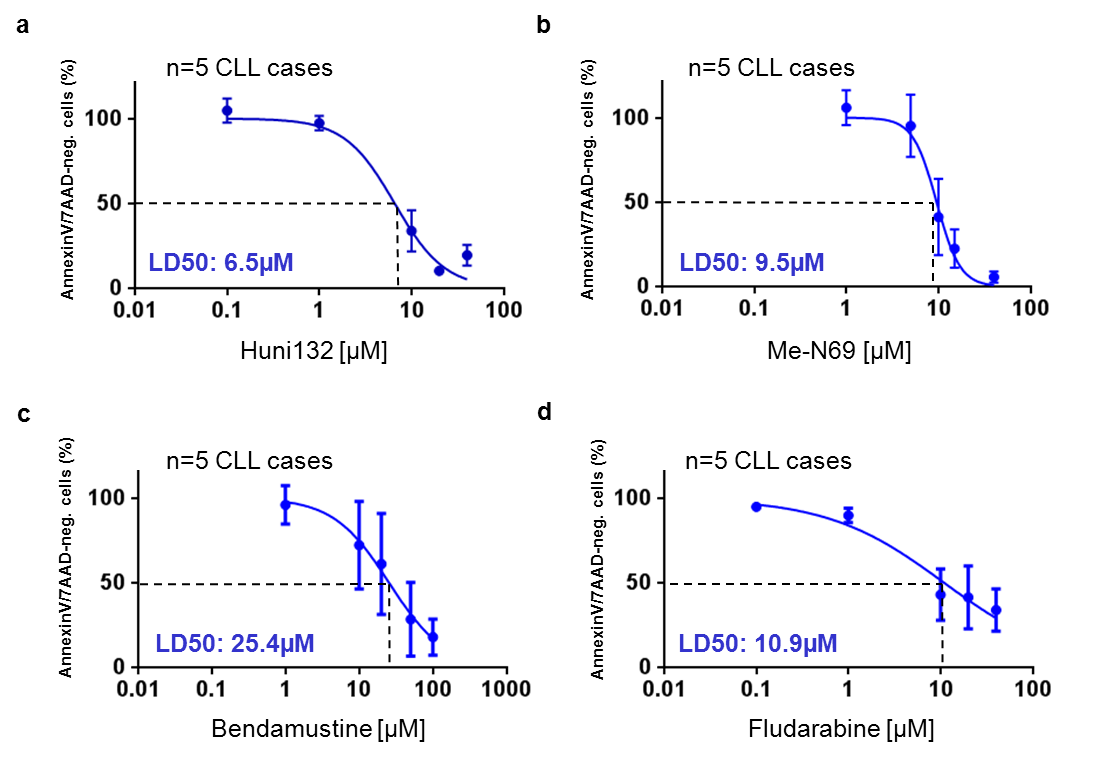
**

Working concentrations of substances **(a-d)** for further in vitro experimentations were determined in a subset of CLL cases based on LD50s (in µM). For that, on day 0, 500 µl of CLL cell suspensions were seeded into 48 well plates (2x10^6^/well) and substances added at the indicated concentrations. Viability / cell death rates were assessed at 24h and 48h by AnnexinV/7AAD flow-cytometric staining. The percentages of AnnexinV/7AAD double-negative cells were normalized to vehicle control and plotted. LD50s were calculated from the dose-response curves via non-linear regression analysis in GraphPad Prism. Shown are the results for 48h to better capture the effects of fludarabine with its slower reaction kinetics. LD50s for all other substances (MCNA and bendamustine) differed not that much between the 24h and 48h time points.

**Figure S2: The B-cell selective cytotoxic profile of the investigated MCNA in comparison to the more T-cell toxic fludarabine is particularly apparent in CLL.**

**
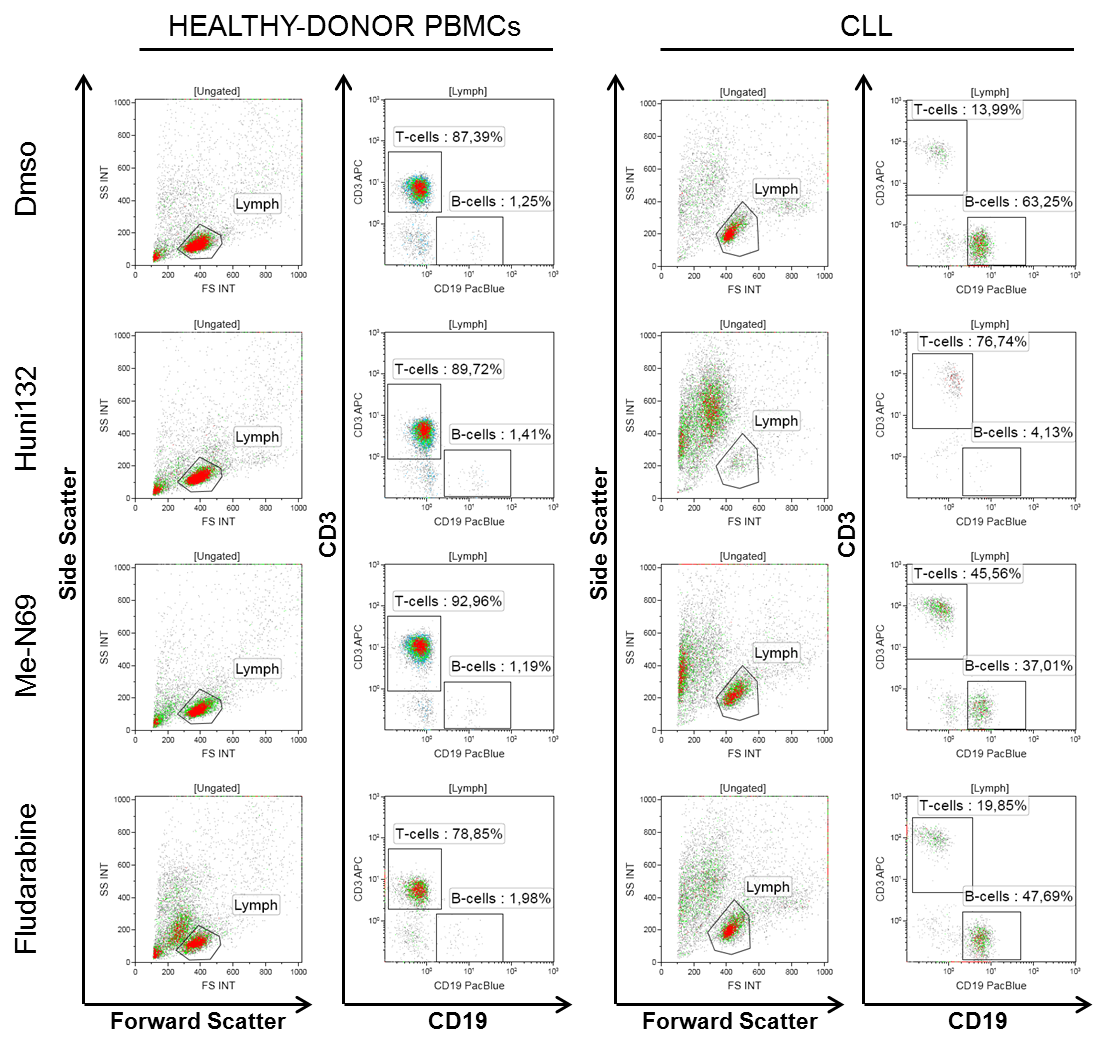
**

To establish the cytotoxic profile of the MCNA towards B-cells and T-cells as compared to the conventional nuceloside analogue fludarabine in more datail (compare to Fig.2A in main manuscript), we preformed additional experiments in healthy-donor derived peripheral blood mononuclear cells (PBMC; n=2) and in CLL samples (n=2). Cells were incubated with MCNA (10μM) and with fludarabine (5μM) for 48h. Flow cytometry histograms of one representative example per group (PBMC, left vs CLL, right) are shown. Verified lymphocytes within the forward/side scatter were differentiated into B-cell and T-cell populations based on CD19/CD3 gating. In contrast to fludarabine, known for its T-cell cytotoxicity, the MCNA show a trend towards a more (malignant) B-cell selective profile. T/B cell ratios for the shown representative samples were as follows; for PBMC: Dmso = 69.9; Huni132 = 63.6; Me-N69 = 78.1; Fludarabine = 39.8; for CLL: Dmso = 0.22; Huni132 = 18.58; Me-N69 = 1.23; Fludarabine = 0.42). These assays work based on the observation that dead lymphocytes no longer membrane-stain for CD3 and CD19, allowing the recording of relative changes within the remainder of the viable lymphocyte population after drug exposure. Furthermore, AnnexinV data (not shown) reveal that the percentage of dying cells (in early apoptosis, yet alive; the AnnexinV-pos. / 7AAD-neg. fraction) within the T-cell gate is higher than in the B-cell gate upon fludarabine exposure and vice versa after MCNA incubation, observed in both healthy-donor derived PBMC and in CLL samples.

**Figure S3: The novel organometallic nucleoside analogues induce marked in-vitro CLL cell death mostly independent of somatic immunoglobulin gene mutation rates and** **previous therapy.**

prior clinical treatment


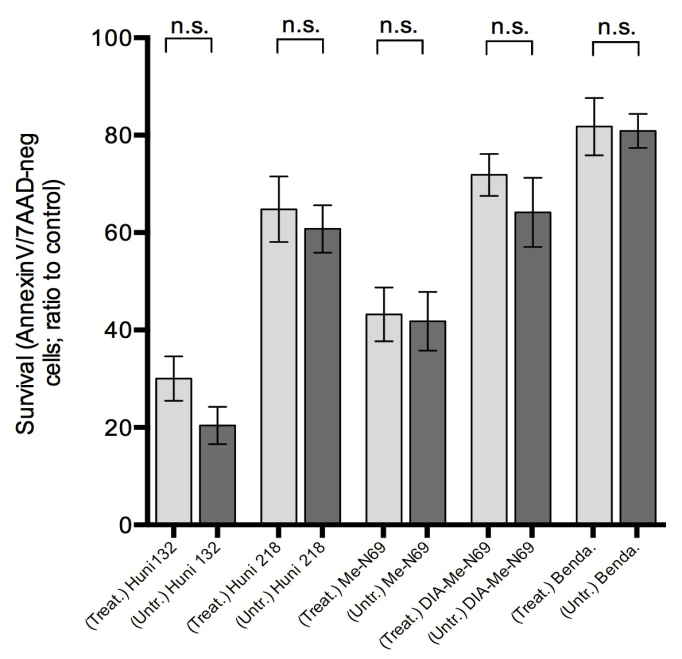


.

IGHV gene mutation status


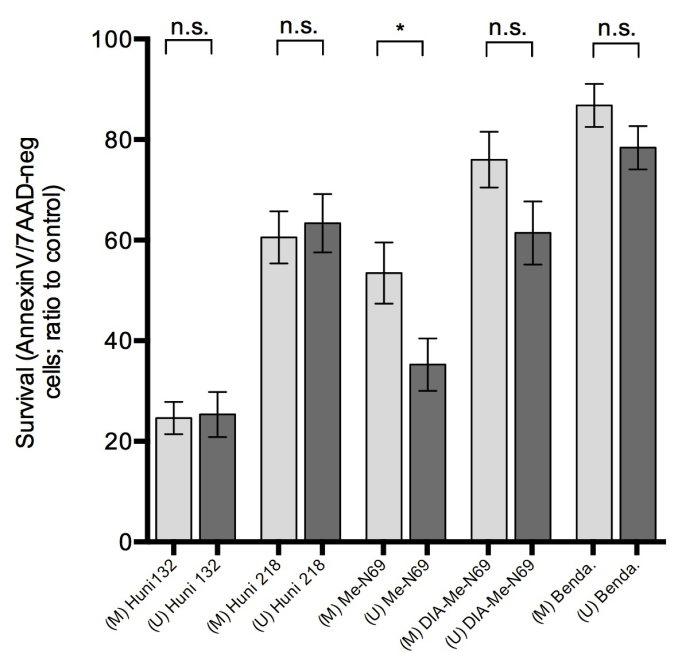


.

**a**  **b**

Incubation (24h) of suspension cultures of primary CLL samples (n=25) with 10μM of all 4 investigated metal-containing nucleoside analogues (MCNA) in comparison to bendamustine (25μM). Cytotoxicity as per AnnexinV/7AAD flow cytometry with means and SEM of surviving cells (AnnexinV-neg. / 7AAD-neg. fraction) charted as ratios to vehicle controls. **(a)** Somatic IGHV gene mutation status did not determine the response to most MCNA. One exeption is Me-N69; here cell death induction in CLL with unmutated IGHV status (U; n=16) is significantly (* P=0.036) higher than in samples from CLL with mutated IGHV genes (M; n=9). **(b)** Overall in vitro substance responses of samples from CLL patients that received any anti-leukemic treatment prior to a time-window of > 4 weeks before use in this study (Treat.; n=13) versus those from previously untreated CLL patients (Untreat.; n=12) show no significant (n.s.) differences in MCNA or bendamustine sensitivity. Lines of treatment are indicated in Table S2 and were mostly fludarabine-containing.

**Figure S4: The MCNA do not compromise NK-tert stromal cell viability.**

**a**

24h

48h

Viability [% rel. to control]

NK-tert bone marrow stromal cells

n.s.

100

50

0


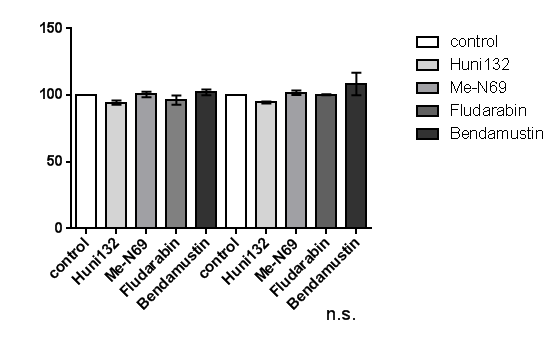


10µM Huni132

10µM Me-N69

10µM Fludarabine

25µM Bendamustine


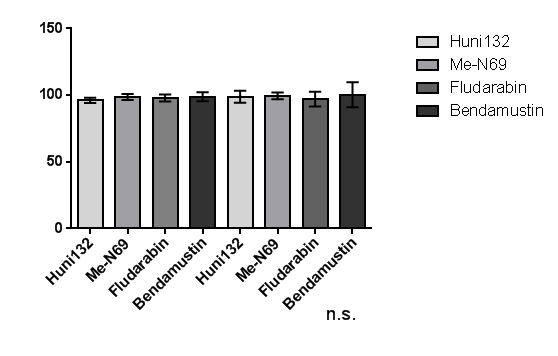


**b**

**Bendamustine**

**Fludarabine**

**Me-N69**

**Huni132**

**Control**

**
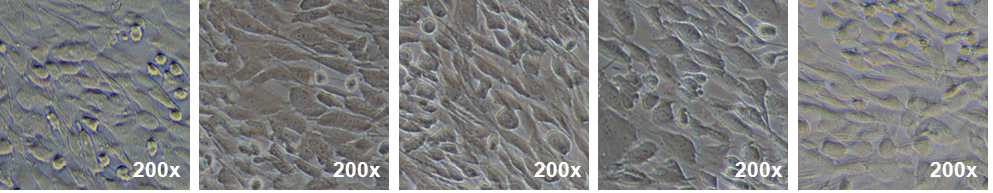
**

To support the assumption that the effect of the MCNA (or fludarabine or bendamustine) on CLL cell viability in the co-culture systems (see Fig.2f) was not attributable to impeding the viability of the supportive stromal component, the human bone marrow stromal cell line NK-tert was exposed to Huni132 and Me-N69 (10μM), fludarabine (10μM), and bendamustine (25μM) for 48h in the absence of CLL cells. Stromal cell viability was quantified by MTT assay (more feasible in these adherent and inter-attached cells than AnnexinV/7AAD flow-cytometry). **(a)** All of the investigated drugs do not significantly (n.s.) reduce NK-tert cell viability (n=3 independent experiments). **(b)** Microscopy (200x) of NK-tert cells after 48h drug treatment reveals no morphologic changes as compared to control (Dmso).

Note that in contrast to our previous modification[1] of the original system[2], we did not employ mitomycine-c based growth arrest of the stromal cells here; hence, they were uncompromised in their capacity to respond to cytotoxic substances.

References:

1. Schrader A, Popal W, Lilienthal N, Crispatzu G, Mayer P, Jones D, Hallek M, Herling M. AKT-pathway inhibition in chronic lymphocytic leukemia reveals response relationships defined by TCL1. Curr Cancer Drug Targets 2014;14(8):700-12.
2. Sivina M, **Hartmann E, Vasyutina E, Boucas JM,** Breuer A, Keating MJ, Wierda WG, **Rosenwald A,** Herling M**,** Burger JA. Stromal cells modulate TCL1 expression, interacting AP-1 components, and TCL1-targeting micro-RNAs in chronic lymphocytic leukemia. Leukemia 2012 Aug;26(8):1812-20.

**Figure S5: Serum components influence MCNA-mediated cytotoxicity of CLL cells in vitro.**

**
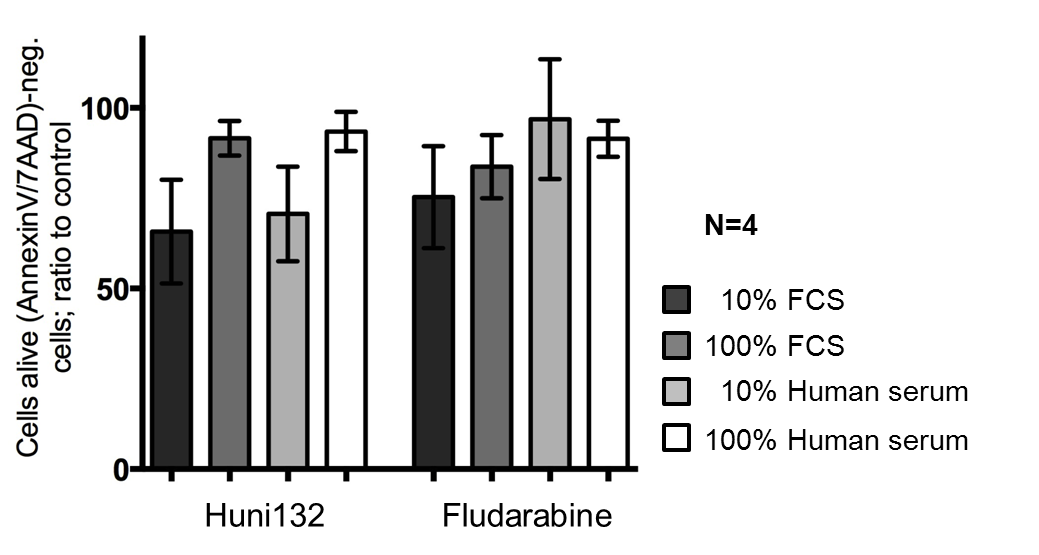
**

In addition to the stromal cell coculture systems (Fig.2f, S4), we investigated the dependence of the CLL cell-death inducing capacity of the most active MCNA Huni132 (10μM) and of the reference nucleoside fludarabine (5μM) on serum concentration and origin. Suspension cultures of n=4 CLL were set up using standard fetal calf serum (FCS; Invitrogen, Carlsbad, California, USA) or human commercial serum (HCS; ‘human serum defibrinated’, Biochrom, Berlin, Germany), both supplemented either at 10% to RPMI1640 medium (Gibco Life Technologies; Carlsbad, California, USA) or as serum alone (at 100%, without RPMI). Huni132 and fludarabine cytotoxicity were assessed by flow cytometry and charted as ratios to control (Dmso). Shown is the AnnexinV/7AAD double-negative fraction with means and SEM. While increasing serum concentrations or the presence of human serum mitigated the cytotoxic effect of fludarabine, only the use of 100% serum of either origin confered some degree of protection from Huni132 mediated cell death.

**Figure S6: The MCNA induce non-necroptotic CLL cell death.**


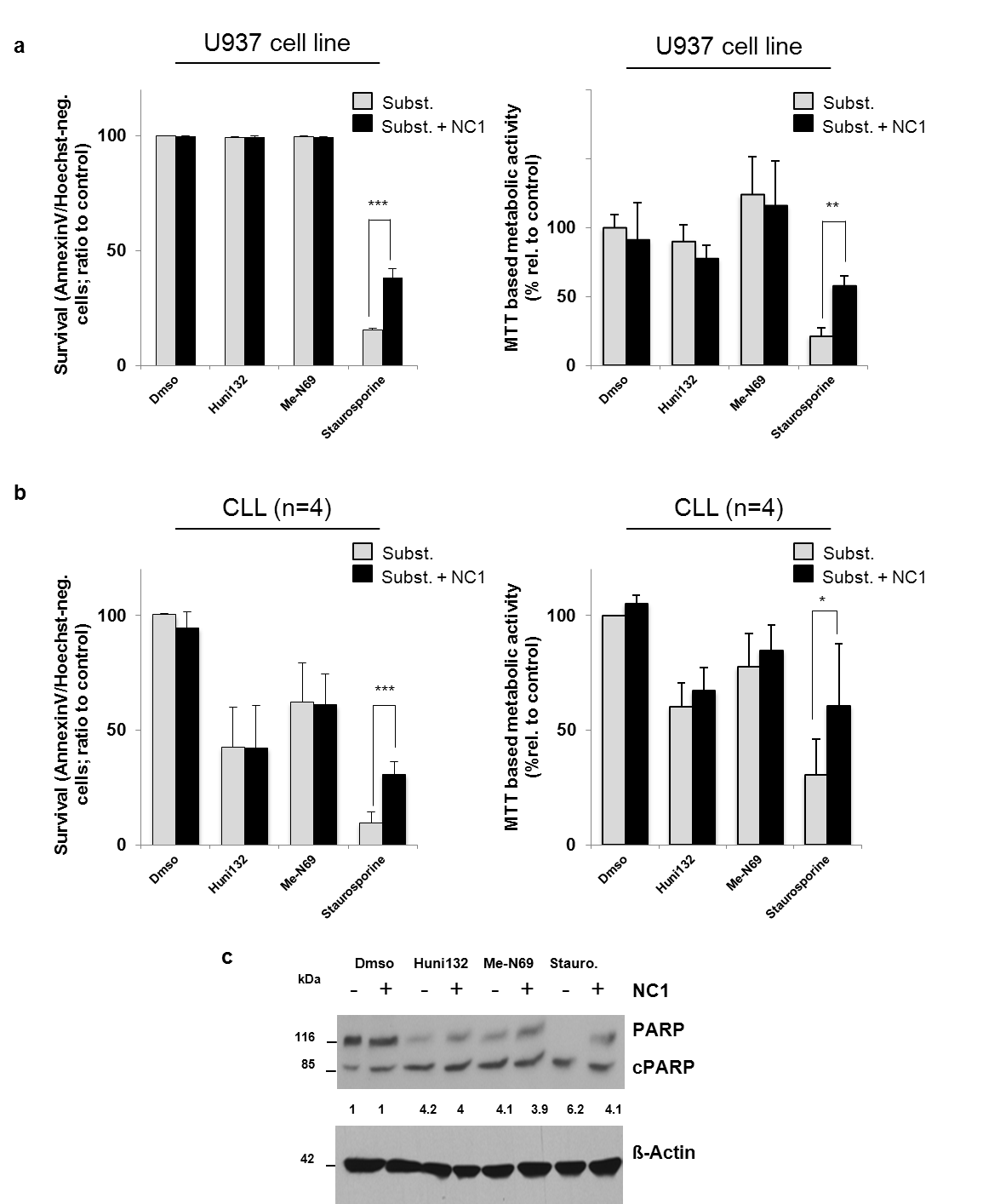


Supplementing the data of Fig.3b of the main manuscript, the cytotoxic effect of the MCNA in suspension cultures of primary CLL samples (n=4) at 24h was studied for Huni132 and Me-N69 (10μM) in comparison to the positive control staurosporine (1μM), all in the presence vs absence of necrostatin-1 (NC1; 20μM). Staurosporine induces apoptosis and necroptosis[1-3], mostly in parallel, but a preferred route is undergone under RIPK1- or caspase-compromised conditions, respectively. As CLL cells may also show some ripoptosome insufficiency[1], U937 myeloid cells, known to be responsive (including via necroptosis) to staurosporine as well[3], were used here as controls. Such induced programmed necrobiosis (necroptosis) should be abrogated by NC1. NC1 is an established potent and selective allosteric inhibitor of necroptosis mostly through targeting the pro-necroptotic receptor-interacting protein kinase (RIPK)[4]. The readouts focus mostly on general cell viability (metabolic activity) as well as on survival / cell death (AnnexinV/Hoechst-double negative fraction), rather than solely on cleavage of PARP, since the latter is a late necroptotic event, dispensible in early necroptosis[3].

**(a)** Left: cytotoxicity as per AnnexinV/Hoechst flow cytometry; means and SEM. NC1 partly abrogates staurosporine-induced cell death (*** P=0.0001) in U937 cells, while it does not do so in the MCNA conditions. Right: the compromised viability (MTT) of U937 cells by staurosporine is partially, but significantly (** P=0.002), rescued in the presence of NC1. **(b)** Left: cytotoxicity as per AnnexinV/Hoechst flow cytometry; mean and SEM. NC1 partly abrogates staurosporine-induced cell death (*** P=0.0001) in 4 cases of CLL, in contrast to the NC1-insensitive marked death-induction by the MCNA. Right: the compromised viability of CLL cells (MTT on 4 cases) by the MCNA is not influenced by NC1 while the reduction of viability by staurosporine is in part rescued by NC1 (*P=0.028). With respect to the evaluations of the specificity of the novel MCNA (main manuscript and Fig.S2), note that the efficacy of the MCNA is marked in the CLL samples while at 10μM hardly any relevant effect is noted in the myeloid U937 cells. **(c)** Western blot analysis (representative of n=4 CLL). The MCNA-triggered PARP cleavage occurs irrespective of NC1 (no major contribution of necroptosis to cell death). In contrast, disappearance of signals for unprocessed PARP after staurosporine is reverted by NC1 (numbers are β-actin corrected densitometric values for cleaved PARP / PARP).

References:

1. Maas C, Tromp JM, van Laar J, Thijssen R, Elias JA, Malara A, Krippner-Heidenreich A, Silke J, van Oers MH, Eldering E: CLL cells are resistant to smac mimetics because of an inability to form a ripoptosome complex. *Cell Death and Disease* 2013, 4:e782.

2. Kitada S, Zapata JM, Andreeff M, Reed JC: Protein kinase inhibitors flavopiridol and 7-hydroxy-staurosporine down-regulate antiapoptosis proteins in B-cell chronic lymphocytic leukemia. *Blood* 2000, 96:393–397.

3. Dunai ZA, Imre G, Barna G, Korcsmaros T, Petak I, Bauer PI, Mihalik R: Staurosporine induces necroptotic cell death under caspase-compromised conditions in U937 cells. *PLoS One* 2012, 7:e41945.

4. Vandenabeele P, Grootjans S, Callewaert N, Takahashi N: Necrostatin-1 blocks both RIPK1 and IDO: consequences for the study of cell death in experimental disease models. *Cell Death and Differentiation* 2013, 20:185–187.

**Figure S7: Diminished MCNA cytotoxicity through PARP inhibition in CLL cells.**

**
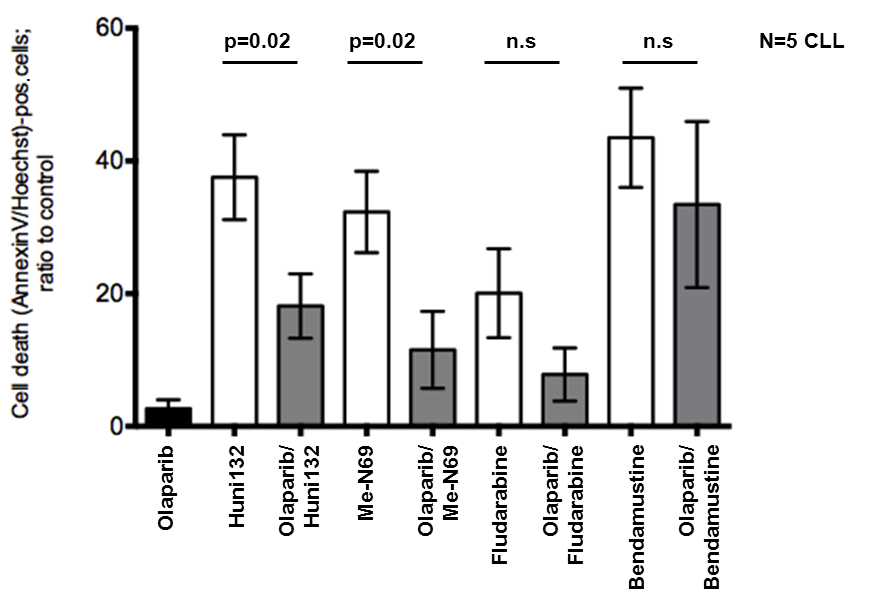
**

The cytotoxic effect ((apoptotic) cell death) of drug treatment in the presence/absence of the PARP inhibitor olaparib (3μM) was assessed after 48h by flow cytometry (AnnexinV/Hoechst; means and SEM). Shown is the percentage of cell death in ATM wild-type (no del11q, no known ATM mutations) primary CLL cells (n=5 cases). PARP inhibition by olaparib effectively protected CLL cells from cell death through the most efficient MCNA Huni132 and Me-N69 (both, p=0.02). This effect was less pronounced for bendamustine and fludarabine. The data indicate the relevant participation of late-stage PARP cleavage in the execution of the MCNA-induced non-conventional apoptotic cell death.

**Figure S8: The programmed cell death induced by organometallic nucleosides is non-autophagic.**


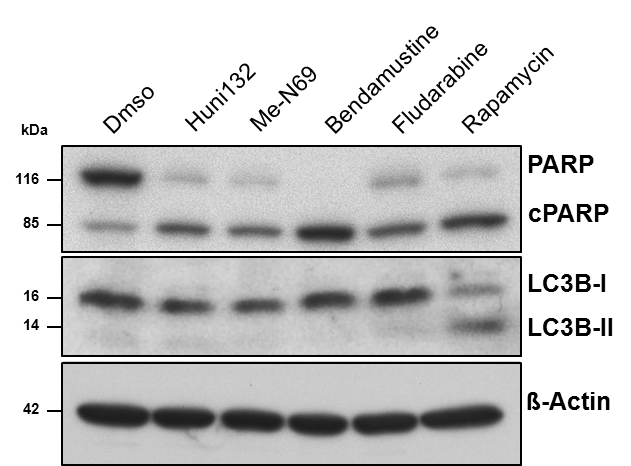


We employed detection of processed LC3B-II levels by immunoblots as an established read-out for induced autophagy[1]. We used CLL cells in suspension cultures (n=3 cases in independent experiments, one representative sample shown). Cells were treated with 2 selected metal containing nucleoside analogues (MCNA; 10µM), bendamustine (25µM), fludarabine (5µM), and rapamycin (20µM). Rapamycin served as a positive control as it induces autophagy through inhibition of TORC1. LC3B-II conversion through rapamycin stood in contrast to unaltered LC3B-II levels that paralleled the PARP cleavage by the 2 most efficient MCNA, by bendamustine, and by fludarabine.

Reference:

1. Barth S, Glick D, Macleod KF: Autophagy: assays and artifacts. *J Pathol.* 2010, 221(2):117–124.

**Figure S9: Rapid mitochondrial depolarization in primary CLL-cells.**


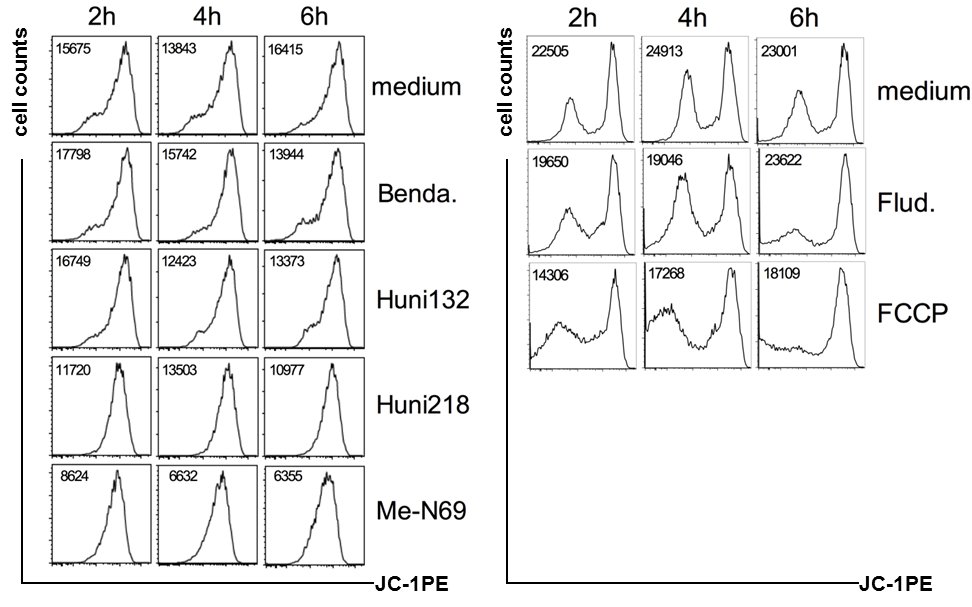


The mitochondrial electrochemical membrane potential (∆ΨM) was semi-quantified in primary CLL-cells (n=3) by flow cytometry using the potentiometric dye JC-1. Shown is one representative sample with the indicated time points. Changes of the JC-1 mean fluorescence index (MFI) were assessed upon treatment with the indicated substances. FCCP (1.5μM) an ionophore uncoupler was used as a positive control for the reduction of ∆ΨM as illustrated by reduced JC-1 staining. Benda. – bendamustine; Flud. - fludarabine. Numbers indicate MFI values. Concentrations were: 25μM for bendamustine, 10μM for all three MCNA, and 10μM for fludarabine. The data supplement Fig.7b of the main manuscript.

**Table S1: Patient demographics and baseline characteristics (summary).**

| **Characteristics** |  |
| --- | --- |
| **All patients, N** | 40 |
| **Age** | N = 40 |
| Median – yrs | 64 |
| Range – yrs | 40 - 86 |
| ≥ 65 yrs – no. (%) | 20 (50) |
| ≥ 75 yrs – no. (%) | 9 (23) |
| **Sex – no. (%)** | N = 40 |
| Male | 29 (73) |
| **Binet stage – no. (%)** | N = 38 |
| A | 16 (42) |
| B | 9 (24) |
| C | 13 (34) |
| **Cytogenetic abnormalities – no. (%)** | N = 31 |
| 17p deletion | 7 (23) |
| 11q deletion | 5 (16) |
| Trisomy 12 | 1 (3) |
| Normal | 4 (13) |
| 13q deletion | 14 (45) |
| **IGHV mutational status – no. (%)** | N = 37 |
| UNM | 18 (49) |
| MUT | 19 (51) |

Abbreviations: yrs: years; no.: number; IGHV: immunoglobulin heavy chain gene variable region; UNM: unmutated; MUT: mutated

**Table S2: Patient demographics and baseline characteristics (by case).**

| **Patient** | **Age** | **Sex** | **Binet Stage** | **Cytogenetics** | **IGHV** | **ZAP70** | **CD38** | **Prior Therapy** |
| --- | --- | --- | --- | --- | --- | --- | --- | --- |
| CLL1 | 51 | m | B | del. 13q | U | neg. | neg. | FCR |
| CLL2 | 68 | m | C | n.d. | U | n.d. | n.d. | C, R-C(O)P |
| CLL3 | 64 | m | A | n.d. | U | pos. | neg. | untreated |
| CLL4 | 48 | m | A | del. 13q | M | neg. | n.d. | n.d. |
| CLL5 | 55 | m | B | del. 13q / del. 11q | U | pos. | pos. | FC, BR, C, L |
| CLL6 | 61 | m | B | n.d. | U | n.d. | n.d. | untreated |
| CLL7 | 60 | m | C | normal karyotype | U | pos. | pos. | FC, RC |
| CLL8 | 79 | m | A | del. 13q | M | n.d. | n.d. | untreated |
| CLL9 | 63 | w | C | del. 11q / del. 17p | U | pos. | pos. | FC, BR, B, R, R-CHOP, L |
| CLL10 | 51 | w | A | n.d. | M | n.d. | n.d. | untreated |
| CLL11 | 72 | m | C | normal karyotype | U | neg. | neg. | untreated |
| CLL34 | 74 | m | A | normal karyotype | M | n.d. | n.d. | F |
| CLL13 | 74 | m | n.d. | compl. | M | n.d. | n.d. | untreated |
| CLL14 | 72 | w | A | normal karyotype | M | neg. | pos. | untreated |
| CLL15 | 58 | m | A | n.d. | M | neg. | neg. | untreated |
| CLL16 | 85 | m | B | n.d. | n.d. | n.d. | n.d. | untreated |
| CLL17 | 70 | m | C | del. 17p | U | pos. | pos. | untreated |
| CLL18 | 66 | w | C | del. 13q | U | pos. | pos. | BR |
| CLL19 | 55 | m | B | n.d. | U | pos. | pos. | BR, L |
| CLL20 | 48 | m | A | n.d. | U | neg. | neg. | untreated |
| CLL21 | 43 | m | A | n.d. | M | neg. | neg. | untreated |
| CLL22 | 66 | m | C | n.d. | M | neg. | neg. | untreated |
| CLL23 | 45 | w | A | del. 13q | M | neg. | neg. | untreated |
| CLL24 | 72 | w | n.d. | Trisomy 12 | n.d. | n.d. | n.d. | FCR |
| CLL25 | 75 | m | A | del. 13q | M | neg. | n.d. | untreated |
| CLL26 | 75 | m | B | del. 13q / del. 17p | U | neg. | n.d. | FC, BR |
| CLL27 | 55 | w | A | del. 13q | M | pos. | n.d. | FR |
| CLL28 | 64 | m | B | n.d. | U | n.d. | n.d. | untreated |
| CLL29 | 59 | w | A | n.d. | M | pos. | neg. | untreated |
| CLL30 | 63 | w | A | n.d. | M | neg. | neg. | untreated |
| CLL31 | 69 | w | B | n.d. | n.d. | n.d. | n.d. | FC |
| CLL32 | 47 | m | A | del. 13q | M | neg. | neg. | FC, R |
| CLL33 | 69 | m | C | n.d. | M | pos. | neg. | C, FA, BR |
| CLL34 | 74 | m | C | del. 13q / del. 11q | M | neg. | neg. | FC, BR, A |
| CLL35 | 74 | m | C | del. 13q / del. 17p | U | n.d. | n.d. | F, FR, BR, CHOP, O, C |
| CLL36 | 67 | m | C | del. 13q / del. 17p | M | n.d. | n.d. | FCR, FC, R, BRL |
| CLL37 | 57 | m | A | n.d. | U | n.d. | n.d. | CV, FC, FCR, R, CHOP, A, L |
| CLL38 | 72 | m | B | del. 11q / del. 17p | M | n.d. | n.d. | C, F, BR, L, FCR |
| CLL39 | 40 | w | C | del. 13q | U | n.d. | n.d. | FC, R |
| CLL40  Abbreviations: B: bendamustine; R: rituximab; F: fludarabine; C: cyclophosphamide; Cl: chlorambucil A: alemtuzumab; L: lenalidomide; CHOP: cyclophosphamide, doxorubicin, vincristine, prednisolone; O: ofatumomab; V: vincristine; n.d.: not defined; prior therapy: anti-leukemic treatment during any time of clinical course with at least a 4-weeks break before sample acquisition; del.: deletion; compl.: complex aberrant karyotype; M: mutated; U: unmutated; neg.: negative; pos.: positive. Fludarabine-refractory disease (cases 32-40) was defined as CLL that did not respond clinically to fludarabine-containing treatments or that recurred within 6 months after therapy with a fludarabine-based regimen; IGHV gene mutation status was determined as described in *Schweighofer C.D. et al, Int J Cancer. 2011 Jun 1;128(11):2759-64.* | 86 | w | C | del. 11q / del. 17p | U | pos. | pos. | F, BR |
